# Establishment of microbial model communities capable of removing trace organic chemicals for biotransformation mechanisms research

**DOI:** 10.1186/s12934-023-02252-6

**Published:** 2023-12-02

**Authors:** Lijia Cao, Sarahi L. Garcia, Christian Wurzbacher

**Affiliations:** 1 Chair of Urban Water Systems Engineering, Technical University of Munich, Garching, Germany; 2 Department of Ecology, Environment and Plant Sciences, Science for Life Laboratory, Stockholm University, Stockholm, Sweden; 3 Institute for Chemistry and Biology of the Marine environment (ICBM), University of Oldenburg, Oldenburg, Germany

**Keywords:** Model communities, Pre-adaptation, TOrCs, Biotransformation

## Abstract

**Background:**

Removal of trace organic chemicals (TOrCs) in aquatic environments has been intensively studied. Some members of natural microbial communities play a vital role in transforming chemical contaminants, however, complex microbial interactions impede us from gaining adequate understanding of TOrC biotransformation mechanisms. To simplify, in this study, we propose a strategy of establishing reduced-richness model communities capable of removing diverse TOrCs via pre-adaptation and dilution-to-extinction.

**Results:**

Microbial communities were adapted from tap water, soil, sand, sediment deep and sediment surface to changing concentrations of 27 TOrCs mixture. After adaptation, the communities were further diluted to reduce diversity into 96 deep well plates for high-throughput cultivation. After characterizing microbial structure and TOrC removal performance, thirty taxonomically non-redundant model communities with different removal abilities were obtained. The pre-adaptation process was found to reduce the microbial richness but to increase the evenness and phylogenetic diversity of resulting model communities. Moreover, phylogenetic diversity showed a positive effect on the number of TOrCs that can be transformed simultaneously. Pre-adaptation also improved the overall TOrC removal rates, which was found to be positively correlated with the growth rates of model communities.

**Conclusions:**

This is the first study that investigated a wide range of TOrC biotransformation based on different model communities derived from varying natural microbial systems. This study provides a standardized workflow of establishing model communities for different metabolic purposes with changeable inoculum and substrates. The obtained model communities can be further used to find the driving agents of TOrC biotransformation at the enzyme/gene level.

**Supplementary Information:**

The online version contains supplementary material available at 10.1186/s12934-023-02252-6.

## Introduction

In recent years, the ubiquitous and frequent detection of trace organic chemicals (TOrCs) in aquatic environments is of increasing concern [1]. Despite their low concentrations of occurrence ranging from a few ng/L to several μg/L, they pose serious adverse impacts on water security and ecosystem health [2]. Wastewater treatment plants (WWTPs) serve as a crucial barrier preventing these contaminants from entering aquatic systems. Although conventional activated sludge and membrane bioreactor technologies were originally designed to remove organic carbon, nitrogen, phosphorus and pathogens, TOrCs are also to some extent removed or transformed [3]. However, more complete TOrC removal requires additional treatment processes such as biodegradation, adsorption, oxidation and ozonation [4–6]. In particular, biodegradation has proven to be a promising approach due to its high removal efficiencies and low energy demand, which is achieved by the microbial communities via metabolism or co-metabolism. For example, TOrCs were degraded more efficiently in the wastewater treatment processes with nitrification, which was related to the activity of ammonia-oxidizing bacteria [7]. Microalgae-bacteria consortium also exhibit advantages in the energy, economy, and environment with great potential in removing various TOrCs [8]. To apply the biodegradation technology in full-scale in the long term and to improve the removal efficiencies, a better understanding of TOrC biotransformation mechanisms is desired. However, the degradation mechanisms remain elusive due to the complexity of microbial interactions occurring in the whole community.

In the last decades, cultivation-independent methodology using next generation sequencing has been developed to explore whole microbial systems [9–11]. However, there are still many challenges with metagenomic analysis [12] and the high complexity in natural microbial communities still prevents us from detangling all microbial interactions and potential ecosystem services. To address this problem, cultivating model microbial communities with low species richness offers a promising opportunity for uncovering specific functions of interest [13, 14]. Simplified communities have been applied to elucidate the microbial community functions and behaviors such as gene regulatory networks, metabolic interactions, and ecological theory [15–17]. For instance, Kang et al. constructed simplified microbial consortia to evaluate keratinous material degradation [18]. The assembled consortia showed similar keratinolytic efficiency to the initial community, showing that simplification can be attained without loss of function and efficiency. Gutiérrez and Garrido determined the key species for the metabolite production in a synthetic consortium of 14 gut microbes during the utilization of prebiotic inulin, providing a basis for defining metabolic roles in the gut microbiome [15].

According to current studies, model communities can be assigned to the categories of synthetic, semi-natural and natural, and they might consist of two-species or higher member complex co-cultures, and enriched model systems [19]. Model communities are usually only designed for specific research purposes and their construction methods can vary across studies. For example, Kang et al. [18] and Garcia et al. [20] used dilution-to-extinction approach to construct model communities. Synthetic communities are often constructed by artificially assembling isolates with the advantage to investigate emergent features that arise from combinations that would not co-exist naturally [21]. Another frequently used method to obtain functional consortia is enrichment that adapt a natural community by exposing it to specific environment conditions (e.g., microbial fuel cells [22]). The enrichment usually leads to microbial composition shift that results from the replacement of sensitive species by tolerant ones upon long-term exposure to chemical stress [23]. In particular, a community adapted to contaminants is enriched in degraders that have great potential in bioremediation [24, 25]. Moreover, synergistic metabolic interactions between species help to stabilize the communities despite fluctuating environmental conditions or even enhance their functions, and these interactions become stronger when the species are distantly related [26]. For example, the interspecies interactions between *Acinetobacter* strain and *Bacillus* strain in a synergistic consortium resulted in higher degradation efficiency of herbicide bromoxynil octanoate than either strain individually [27].

In this study, we aim to establish new model communities that can be applied to mechanistically investigate biological TOrC transformation processes and to help improve biotransformation technologies. A combination of enrichment steps and the dilution-to-extinction method were tested in order to provide a robust, standardized protocol for obtaining diverse model communities that can biotransform TOrCs. We hypothesized that (i) the pre-adaptation process will benefit the selection of model communities in terms of diversity, and (ii) the phylogenetic diversity of the resulting model communities will influence the TOrC removal rates. We followed a series of enrichment steps to adapt the inoculum to TOrC environment and subsequently used the dilution-to-extinction approach to establish thousands of potential model communities. We also tested the effects of the inoculum and its initial diversity on the protocol in order to provide a universal framework for several starting communities for TOrC biotransformation research.

## Materials and methods

### TOrC selection, inocula sampling and growth medium preparation

A broad range of TOrCs with different properties and uses are present in WWTP effluents [28]. To adapt microbial communities to diverse chemicals, 27 TOrCs were selected for this study based on their uses, occurrence and biodegradability. Pharmaceuticals, industrial chemicals and artificial sweeteners with worldwide high consumption rate and frequent detection in aquatic environment were included. Moreover, the detected concentrations of TOrCs in water and wastewater was considered. In addition, the biodegradability indicated by the biotransformation efficiencies was an important selection criterion as well as their potential ecological risks. A complete list of selected compounds including the uses, biotransformation efficiencies, occurrence in aquatic systems, and ecological risks assessed by risk quotients (RQ) [29] can be found in Additional file 1: Table S1.

27 TOrCs were prepared as a mixed stock solution at the concentration of 50 μmol/L per compound. Samples for inoculation were obtained from tap water (Garching, Germany), technical sand treated with tertiary effluent (Garching, Germany), soil (Garching, Germany) and sediment (47° 47′ 16" N, 11° 18′ 16" E, Osternsee, Germany) on November 2021. Samples were immediately taken to the lab for storage at 4 °C until usage. Sediment was collected by a gravity corer and was divided as sediment surface (0 m) and sediment deep (0.3 m). Growth medium was prepared with mineral salts (1L: 448 mg Na_2_HPO_4_·2H_2_O, 746 mg KH_2_PO_4_, 70 mg MgSO_4_, 120 mg (NH_4_)_2_SO_4_, 1 mg Ca(NO_3_)_2_, 0.1 mg H_3_BO_3_, 2.5 mg FeSO_4_·7H_2_O, 0.75 mg MnSO_4_·H_2_O, 1.3 mg ZnSO_4_·7H_2_O, 0.25 mg CuSO_4_·5H_2_O, 0.3 mg Co(NO_3_)_2_·6H_2_O, 0.15 mg Na_2_MoO_4_·2H_2_O, 0.01 mg NiSO_4_·7H_2_O) [30] and additional TOrCs mixture as the sole carbon source with a final concentration of 5 nmol/L per compound within the range of aquatic environmental occurrence [4]. Mineral salt medium was sterilized by autoclaving (120 °C, 21 min), and TOrCs solution was filtered twice by 0.22 μm Sterivex filter (Millipore).

### Pre-adaptation to increasing concentrations of TOrCs

Each sample was used as inoculum (50 g of sediment, sand and soil, and 50 mL of tap water) and was directly added into 450 mL growth medium in a 1 L sterile glass bottle in triplicates, resulting in 15 bottles in total. They were incubated in stationary in the dark at room temperature for one month. Afterwards, 450 mL suspension was discarded and replaced by fresh growth medium. The pre-adaptation process consisted of six phases which took six months. TOrC concentrations were adjusted in each phase (P1: 50 nmol/L, P2: 500 nmol/L, P3: 1000 nmol/L, P4: 1000 nmol/L, P5: 2500 nmol/L, P6: 50 nmol/L). This process was designed as a selective enrichment to let microbial communities adapt increasingly to TOrCs (the sixth phase with 50 nmol/L for recovery) and to decreasing alternative sources of carbon. Furthermore, to avoid dramatic environmental perturbations, TOrC concentrations increased gradually from 50 to maximum 2500 nmol/L. Despite the increasing selection pressure brought by TOrCs, the variation of concentrations was still in the range of real-world conditions (Additional file 1: Table S1). After six passages, the natural carbon source in P0 was diluted 10^–6^, making it possible to determine the removal of TOrCs as the only carbon source in P6. In the final step P6, the aim was to get close-to-natural conditions of TOrC concentrations, as an adjustment to the final concentration of the degradation assays. The pre-adapted microbial communities from P6 were used as the inocula for the dilution-to-extinction steps setting up the model communities.

### Cell counting

Cell counts were measured at the end of each phase to ensure the growth of microorganisms. 50 μL well mixed cell samples were directly stained with 5 μL of 2.5 μM nucleic acid stain SYTO 13 (Invitrogen) for 10–15 min at room temperature in the dark [31]. Samples were then added into the wells of a 96 flat well plate and loaded into the flow cytometer (CytoFLEX, Beckman Coulter, Germany) for counting. Stained bacterial cells excited at 488 nm were enumerated in the FITC channel, and background noise of both water and particles were gated out.

### Dilution-to-extinction

Microbial communities from adapted (P6 community; triplicates were pooled) or non-adapted (P0 community; triplicates were pooled) inoculum were serially diluted to 0, 0.1, 1, 10, 50, 100, 200, 500, 800, 1000, 2500, 5000, 10,000 cells/mL using growth medium spiked with 5 nmol/L TOrCs mixture in 96 deep well plates to establish model communities. The P6 inocula were only diluted from 0 to 1000 cells/mL, since we expected a more constant initial diversity. 48 wells of a 96 deep well plate were inoculated with 1 mL suspension from each dilution, resulting in 4704 wells from all dilutions and all inocula, and 16 blank wells (without cells) as control. All plates were incubated in the dark at room temperature for 21 days, which was shown to reach the stable growth phase according to our pre-experiment. After 21 days, cell counting was used to screen the wells for positive growth defined as a minimum of 1 × 10^6^ cells/mL (the ubiquitous density observed in aquatic environment) [20, 32, 33].

### TOrC degradation experiment

Microbial communities with different diversity were diluted to 1000 cells/mL, and then 1 mL suspension was inoculated in a new plate spiked with 5 nmol/L TOrCs to investigate their removal rates (i.e., TOrC degradation percentage) within 21 days. Similarly, all P6 inocula and blanks (TOrC medium without cells and with autoclaved cells) were incubated in the plates for the same period. Blanks acted as controls to evaluate the abiotic TOrC degradation. Cell counts were monitored at 2, 5, 7, 8, 12, 14, 16, 19, 21 days to estimate the growth rates and final cell counts. TOrC concentrations were measured at the start (0 d) and end (21 d) time point by liquid chromatography coupled with tandem mass spectrometry (LC–MS/MS) following Müller et al. [34]. Briefly, 200 μL of samples collected from pre-adaptated P6 were firstly diluted 10 times to meet the requirements for detection limit (≤ 10 μg/L). 1 mL of samples collected from biodegradation experiment were diluted 2 times to obtain enough volume for measurement. 1900 μL of diluted samples were mixed with 100 μL of internal standard, and then filtered through 0.22 μm polyvinylidene difluoride (PVDF) membrane filters into 2 mL amber glass vials. Samples were performed on a PLATINblue UPLC system (Knauer, Germany) equipped with a Phenomenex Kinetex PFP 100-Å chromatographic column (150 × 3 mm, 2.6 μm). The mobile phase was composed by a gradient of Milli-Q water and LC–MS grade methanol (Merck, Germany), supplemented with 0.1% formic acid. The used mass spectrometer was a SCIEX triple Quad 6500 equipped with a Turbo V ion source for electrospray ionization.

### DNA extraction, library preparation and sequencing

Samples of the initial inocula P0, adapted inocula P6 and final model communities were subjected to DNA extraction by using the DNeasy PowerSoil Pro Kit (QIAGEN) according to manufacturers’ instruction. Extracted DNA was quantified using dsDNA Broad Range Assay (DeNovix, USA) in a fluorometer (DeNovix, USA). 16S rRNA gene was amplified using the 27F (AGAGTTTGATCMTGGCTCAG) and 1492R (TACGGYTACCTTGTTACGACTT) primers. The PCR mix included 25 μL 2 × GoTaq® colorless master mix (Promega), 1 μL of each primer (20 μM), 1 μL DNA template, and 23 μL nuclease-free water. Thermocycling conditions included an initial denaturation at 95 °C for 2 min, 30 cycles of denaturation for 1 min, annealing at 56 °C for 1 min and extension at 72 °C for 2 min, and a final extension for 10 min. The PCR products were checked for quality by a 1.5% agarose gel electrophoresis and purified using MagSi-NGS^PREP^ Plus beads (Steinbrenner, Wiesenbach, Germany) and AMPure XP beads (Beckman Coulter, Germany) according to the manufacturer's instructions. Library preparation was performed using native barcoding kit 96 (SQK-NBD112.96, ONT) following the manufacturer's instructions. A total of 75 μL library pool was loaded into an Oxford Nanopore R10.4 flow cell and sequenced with MinION™ Mk1C device. The run was stopped once desired number of reads (around 15,000 reads per sample) were achieved.

### Amplicon sequencing data processing

Raw fast5 reads generated by MinION were basecalled with high accuracy model and converted to FASTQ files using Guppy v3.6.0 (https://timkahlke.github.io/LongRead_tutorials/BS_G.html). Afterwards we processed the amplicons following Cuscó et al. [35] and Karst er al. [36]. Sequencing adapters were removed using Porechop v0.2.4 (https://github.com/rrwick/Porechop). Sequences were then trimmed using NanoFilt v2.8.0 with the parameters of -q 9 and -l 1000 [37]. Trimmed FASTQ files were converted to FASTA files using SeqKit v2.3.0 [38]. Processed reads were clustered into operational taxonomic units (OTUs) at 90% identity to adjust for the higher sequencing error of Nanopore reads using USEARCH v11 [39] and VSEARCH v2.22.1 [40]. To calculate the relative abundances of OTUs, all reads before quality trimming were mapped to OTU sequences at 90% identity by VSEARCH. Singleton OTUs with only one read were discarded. Generated OTU table was further filtered by a cutoff of 1% read abundance per sample. Taxonomy assignment of OTUs was performed by USEARCH SINTAX algorithm [41] against the RDP database v16 [42] with 0.8 similarity cutoff. Phylogenetic tree file was also generated by USEARCH.

### Statistical analysis

Filtered OTU table was imported into R v4.2.1 for alpha diversity calculation and phylogenetic diversity comparison. Reads were rarefied to even depth for calculating Shannon index, Simpson index and species richness by R package vegan v2.6.4 [43] and phylogenetic diversity by R package picante v1.8.2 [44]. We used *t*-test to evaluate significant differences of microbial diversity between pre- and non-adaptation groups. Non-metric multidimensional scaling (NMDS) analysis was performed based on generalized unifrac distance with alpha setting of 0.5 by R package GuniFrac v1.7 [45]. Significance between groups was determined by a permutational multivariate analysis of variance (PERMANOVA). Principal coordinate analysis (PCoA) of TOrC removal performance based on Euclidean dissimilarities was conducted to find clusters of similar groups of samples. Ten persistent TOrCs (i.e., amisulpride, antipyrine, candesartan, fluconazole, iopromide, primidone, tramadol, trimethoprim, venlafaxine, 4-formylaminoantipyrine) that were not degraded in the experiment were removed from the dataset prior to the analysis. Correlations of diversity, cell counts, average removal with community dissimilarities were performed by R package vegan using the function Envfit and Adonis. The cor.test function in R was used to test the relationship between phylogenetic diversity and TOrC removal performance in terms of removal rates and removal diversity (number of TOrCs above 20% simultaneous removal).

## Results

### Overview on the establishment and selection of model communities

**Fig. 1:**
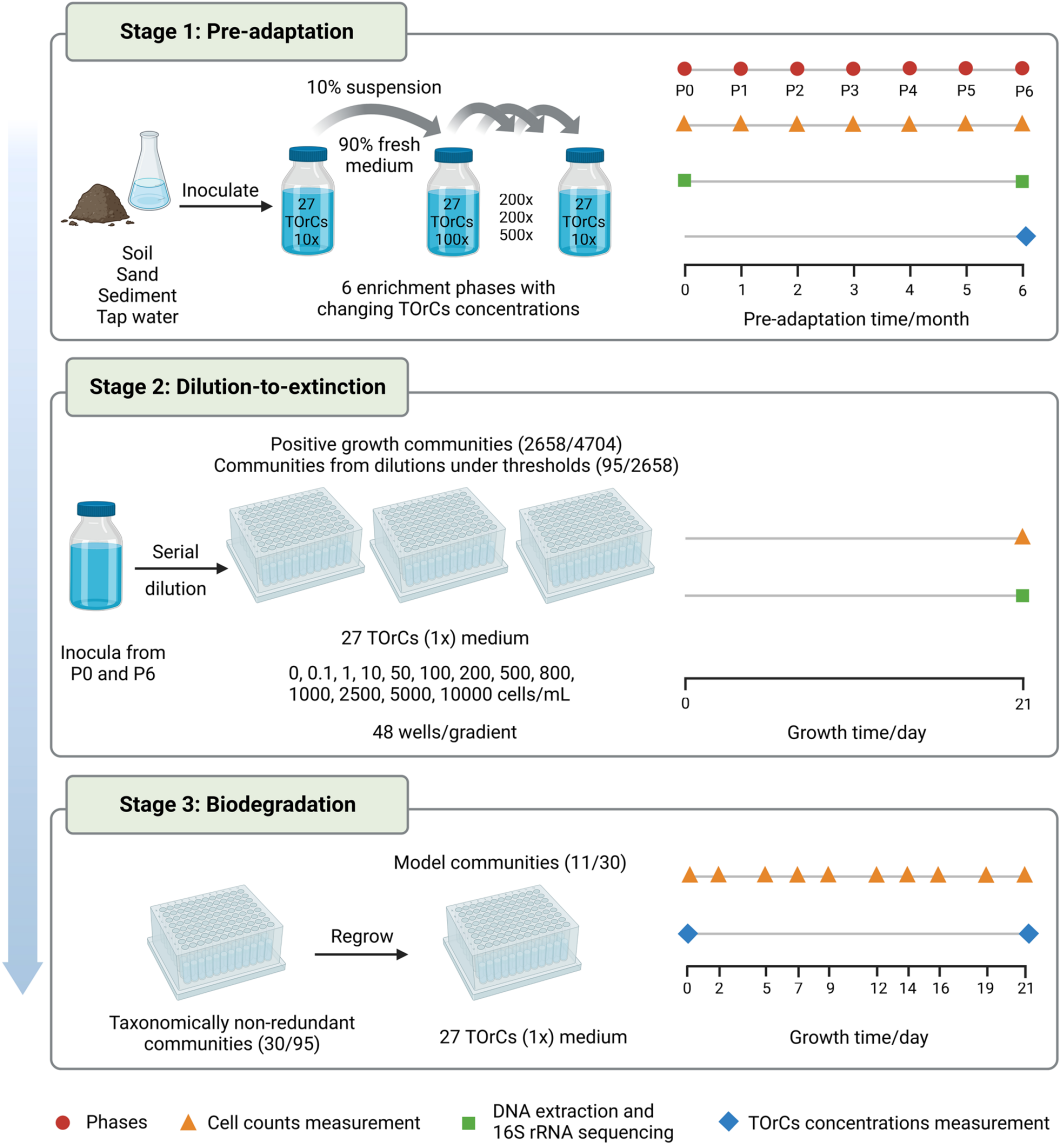
Workflow of model communities establishment. There were 3 stages with inoculum pre-adaptation (stage 1), dilution-to-extinction and microbial communities regrowth (stage 2), and TOrC biodegradation experiments for selecting model communities (stage 3). Data in parenthesis (stage 2 and 3) showed the number of selected communities/all communities derived from non-adapted P0 and pre-adapted P6 inocula

We established model communities that can grow on TOrCs by standardizing a workflow (Fig. 1) that can provide a basis for similar studies with exchangeable organic substances. Overall, there were three stages consisting of pre-adaptation, dilution-to-extinction, and biodegradation that have been explained in the materials and methods section. During the pre-adaptation process (stage 1, Fig. 1), cell densities were measured at the end of each phase confirming a certain inhibition effect of TOrCs at concentrations higher than 50 nmol/L on cell growth by on average one order of magnitude, excluding tap water and sand communities (Additional file 2: Fig. S1). Taxonomies were assessed for P0 and P6 to monitor the influence of TOrC selection pressure on the diversity of the whole community (results were shown in the following section).

**Fig. 2:**
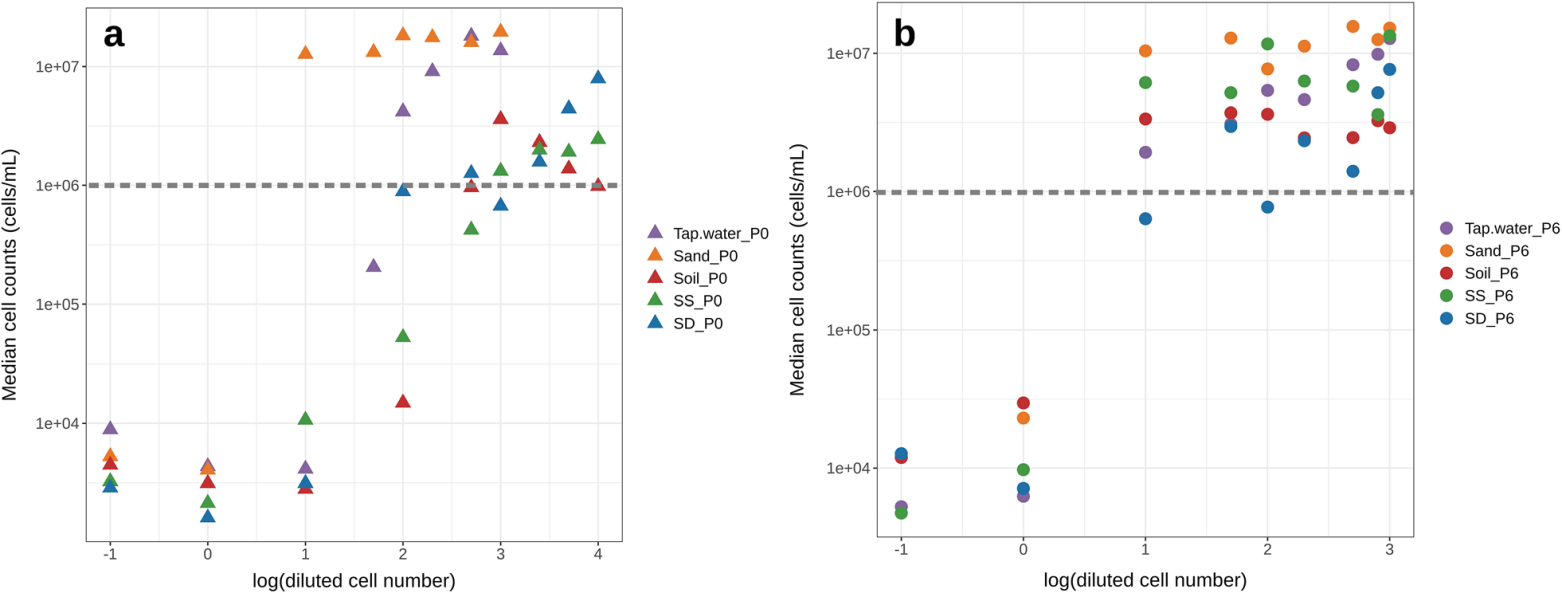
Median cell counts of microbial communities growing from different diluted cell numbers of (**a**) non-adapted P0 and (**b**) pre-adapted P6 inocula after 21 days incubation, n = 48. Grey dash lines represent the value of 1 × 10^6^ cells/mL

After pre-adaptation, TOrC degradation by P6 communities was measured and a dilution-to-extinction experiment was conducted (stage 2, Fig. 1) in parallel with non-adapted P0 communities. There were 4704 incubated wells in total, of which 2658 showed positive growth (cell densities ≥ 1 × 10^6^ cells/mL) after the first 21 days incubation. The median cell counts of all diluted communities after 21 days incubation in stage 2 point to a putative sigmoid-like function that indicates an inoculum type specific threshold (Fig. 2). We used this data to determine an inoculum specific threshold, which we defined as the initial inoculated cell number per well that resulted in nearly 100% wells with positive growth. In the non-adaptation group, the initial inoculum had largely varying dilution thresholds. Microbial communities from tap water, sand, soil, sediment surface and sediment deep reached 100% growth at the dilution of 100, 10, 500, 1000, 100 cells/mL, respectively (Fig. 2a). In contrast, in the pre-adapted group, 10 cells/mL dilution led to growth in all wells independent from any of the five tested inocula (Fig. 2b). This result signified a standardization effect of the pre-adaptation process on initial inoculum with varying diversity. Among the 2658 positive growth wells, we selected 45 wells from non-adapted (n = 9 per inoculum) group and 50 wells from pre-adapted group (n = 10 per inoculum). The well selection was based on three criteria. Firstly, their initial cell numbers were below the above described dilution threshold. Around 30% of the wells showed positive growth below the respective inocula specific dilution thresholds, indicating potential model communities of low complexity (Additional file 2: Fig. S2). Secondly, the minimum well number was applied to all inocula in the same treatment group to keep the sample size identical for statistics. Thirdly, when there were more than the minimum number of positive growth wells, we selected the highest cell density wells evenly from different initial cell numbers (e.g., 1, 10, 50, 100 cells/mL) to include potentially diverse richness communities.

In the next step (stage 3, Fig. 1), we characterized the 95 selected communities by using DNA based amplicon sequencing, which allowed us to screen for taxonomic redundancies. The taxonomic classification based on the 16S rRNA gene resulted in 259 filtered OTUs across all 95 communities. The 95 microbial communities exhibited a median richness of 10, spanning 2–32 OTUs. From these communities, we identified 30 taxonomically non-redundant model communities (15 from non-adapted P0 and 15 from pre-adapted P6), which we subjected to a biodegradation experiment to test the influence of the taxonomic composition in the TOrC biotransformation and to assess their overall removal performance. Finally, 19 communities degraded one to four compounds by 20–100% (atenolol, ibuprofen, hydrochlorothiazide, gemfibrozil, climbazole, sulfamethoxazole), the other 11 communities achieved better removal performance on 5 to 9 compounds by above 20% (atenolol, ibuprofen, hydrochlorothiazide, gemfibrozil, climbazole, sulfamethoxazole, benzotriazole, caffeine, carbamazepine, diclofenac, gabapentin, 4/5-methylbenzotriazole) (Additional file 2: Fig. S3). These 11 communities were regarded as model communities of interest for future research, which grew well, had low but different taxonomic diversity with two to eleven genera per community, and removed diverse TOrCs (Additional file 2: Fig. S4).

### Pre-adaptation affected the diversity of whole community and model communities

**Fig. 3:**
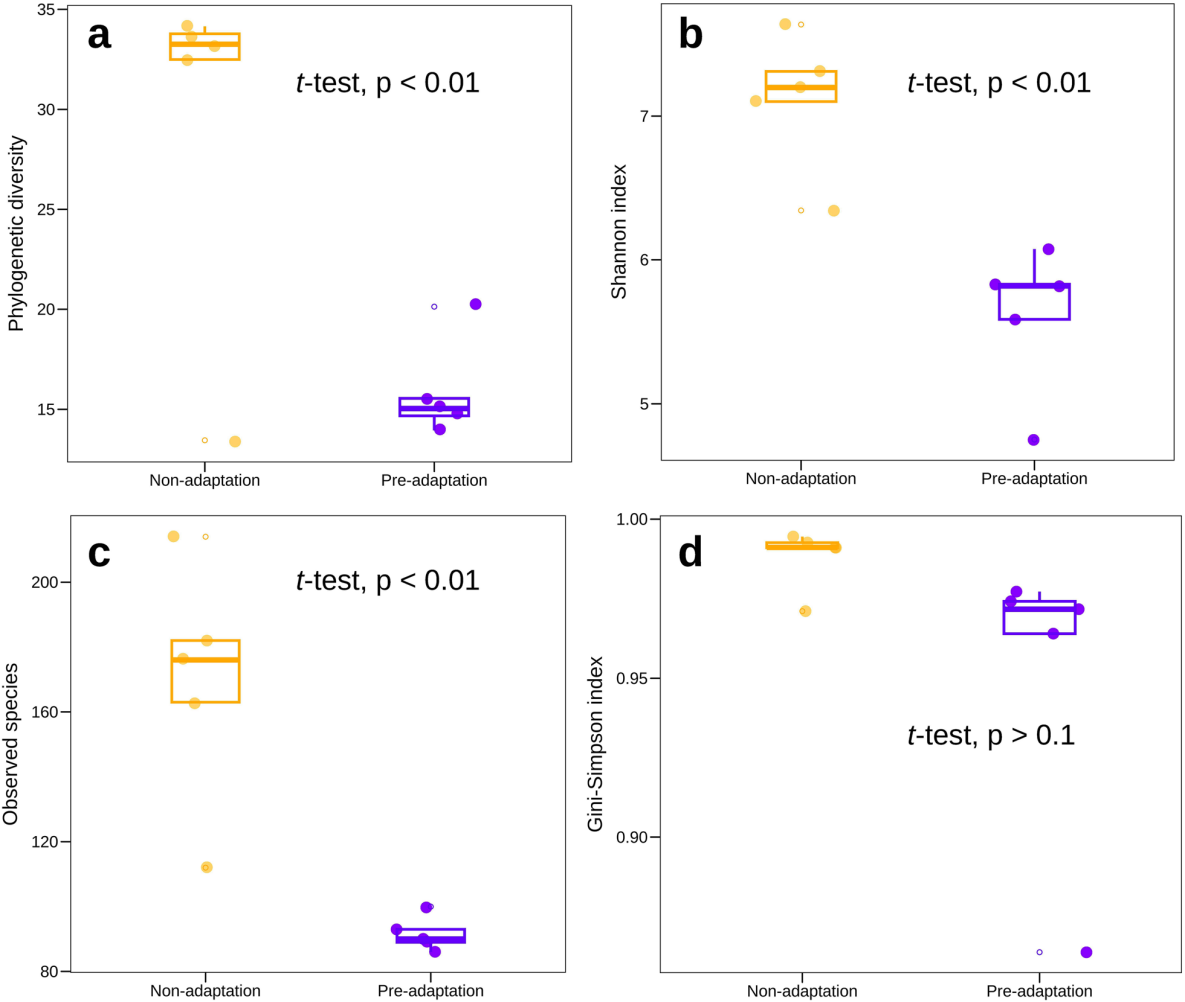
Diversity of pre- and non-adapted inocula in respect of (**a**) phylogenetic diversity, (**b**) Shannon index, (**c**) observed species and (**d**) Gini-Simpson index

From the dilution-to-extinction experiment, we could clearly observe differences in the initial cell numbers per well that leads to successful growth of the model communities in the presence of TOrCs (Fig. 2). One explanation is that the different microbial communities from different environments have varying diversity, and different number of taxa that can grow on or survive the concentrations of TOrCs tested. When analyzing the taxonomic composition of P0 and P6 communities, we could clearly notice a reduction in diversity in terms of phylogenetic diversity (Fig. 3a), Shannon diversity (Fig. 3b), and observed richness (Fig. 3c). For instance, the observed species for the pre-adapted P6 inocula ranged from 86 to 112 species, while the original P0 inocula contained 163–214 species (Fig. 3c). An exception was observed for the sand inoculum (shown as an outliner), where the adapted community diversity was similar to the non-adapted community diversity. P0 and P6 had no significant difference of evenness (Fig. 3d), indicating the microbial communities still distributed evenly after pre-adaptation.

**Fig. 4:**
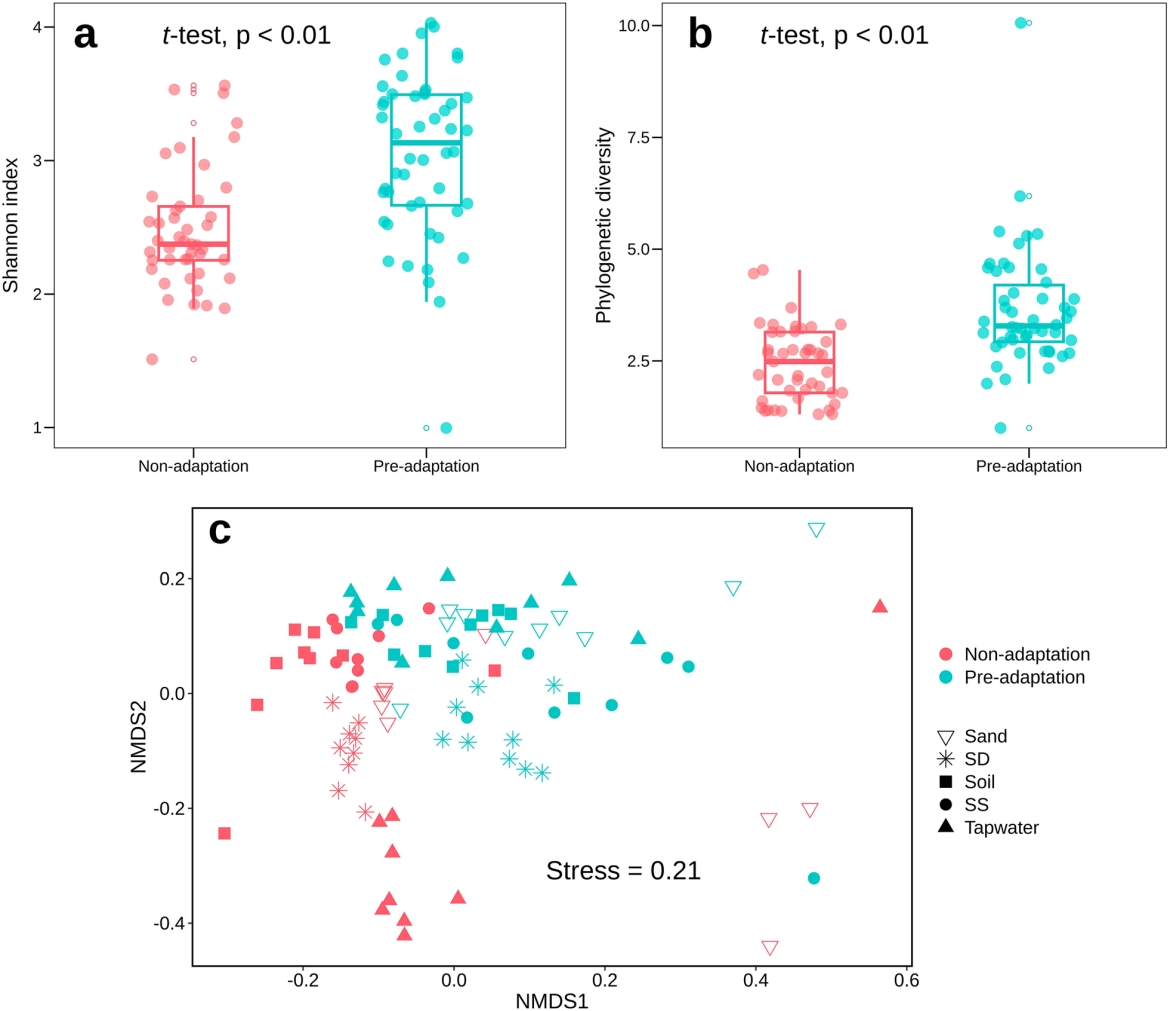
Diversity of 95 microbial communities derived from pre- and non-adapted inocula. (**a**) Shannon index, (**b**) phylogenetic diversity, (**c**) NMDS plot showing beta diversity based on GUniFrac with alpha 0.5 (stress value = 0.21). PERMANOVA test was performed on combined GUniFrac with alpha 0, GUniFrac with alpha 0.5 and weighted UniFrac

On the contrary, the observed reduced richness of P6 inocula had an opposite effect on the resulting model communities, i.e., the model communities from the pre-adapted inocula had greater richness in respect of Shannon diversity (Fig. 4a) and phylogenetic diversity (Fig. 4b) than the model communities derived from non-adapted inocula.

**Fig. 5:**
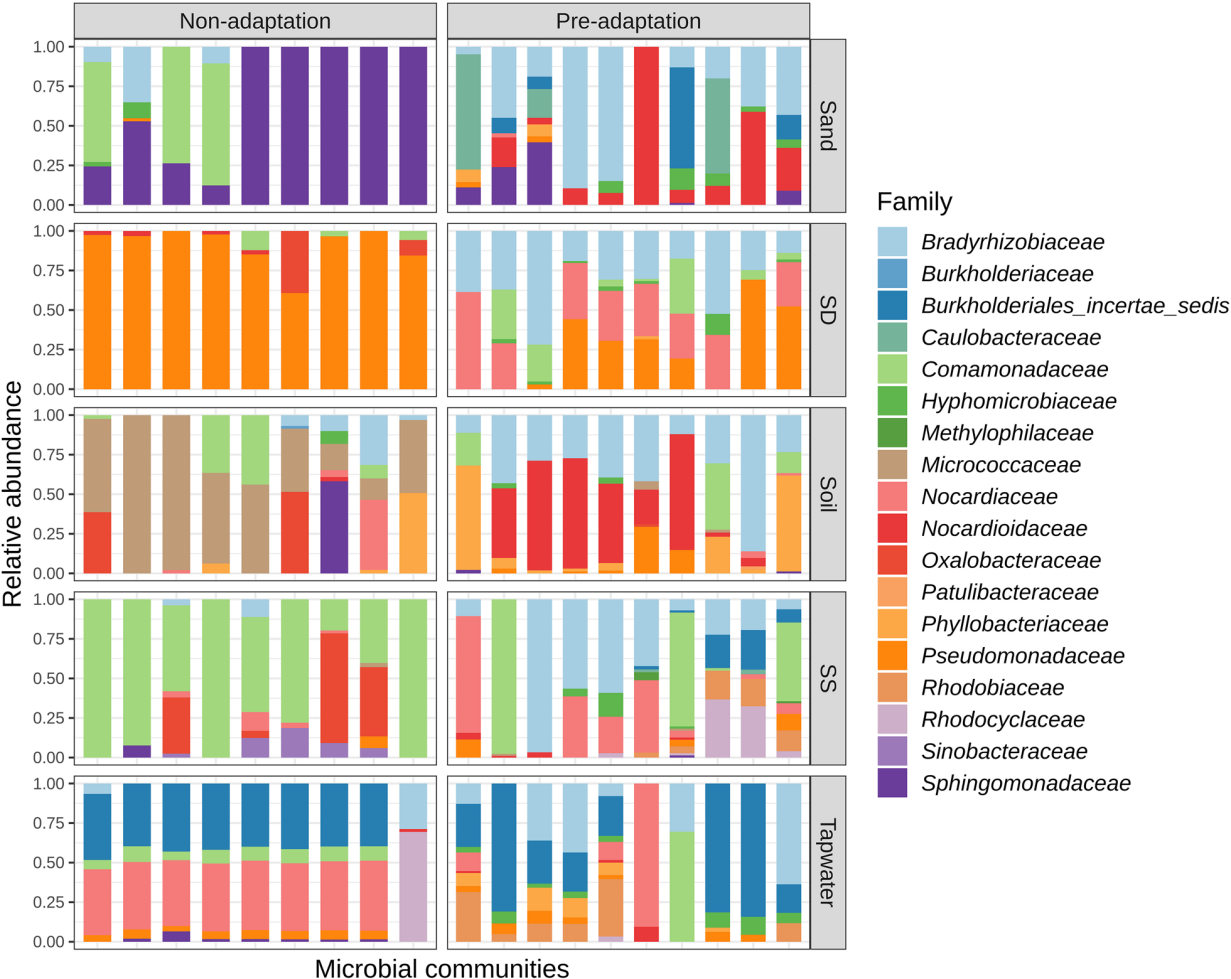
Comparison of microbial structure of 95 potential model communities derived from pre- and non-adapted inocula at the family level

A closer look at the microbial community structure of the 95 microbial communities revealed that communities from pre- and non-adaptation groups were differently structured according to Unifrac distance (PERMANOVA, R^2^ = 0.075, p = 0.01) (Fig. 4c). Microbial communities from non-adapted tap water inoculum (n = 9) together with several communities from non-adapted and pre-adapted sand (n = 5) were most distinct in relation to the other communities. The observed grouping of model communities belonging to pre- and non-adapted inocula was also reflected in the taxonomy at the family level (Fig. 5). In the non-adaptation group, most model communities were dominated by one or two families (e.g., non-adapted sediment deep communities were dominated by 91% *Pseudomonadaceae* in average). In the pre-adapted group, species distributed more evenly (e.g., *Bradyrhizobiaceae*, *Nocardiaceae*, *Pseudomonadaceae* and *Comamonadaceae* composed the communities from pre-adapted sediment deep by 34%, 28%, 25%, and 11% on average, respectively) while maintaining some of the taxa that were also dominant in the model communities from the non-adapted inocula (e.g., *Pseudomonadaceae, Nocardiaceae, Comamonadaceae*). Some rare taxa e.g., *Caulobacteraceae* and *Micrococcaceae* occupying less than 2% in all inocula were abundant in the pre- and non-adapted model communities (18% and 73% of *Caulobacteraceae* in two model communities from adapted sand; 14–100% *Micrococcaceae* in all model communities from non-adapted soil).

We hypothesized that exposing whole communities to TOrCs allows the microbial community to go through a succession. In this succession, naturally abundant but vulnerable to TOrCs microorganisms reduce in numbers while allowing rarer microorganisms to slowly increase in numbers accompanied by members of the community that could act as cornerstones of resilience (i.e., the key taxa that maintain the stability and recover the functions of a community). Hence, pre-adaptation resulted in an overall reduced richness of the whole community while having a positive effect on the species that can grow on or survive TOrCs and could then thrive in the model communities.

### TOrC removal performance by model communities

**Fig. 6:**
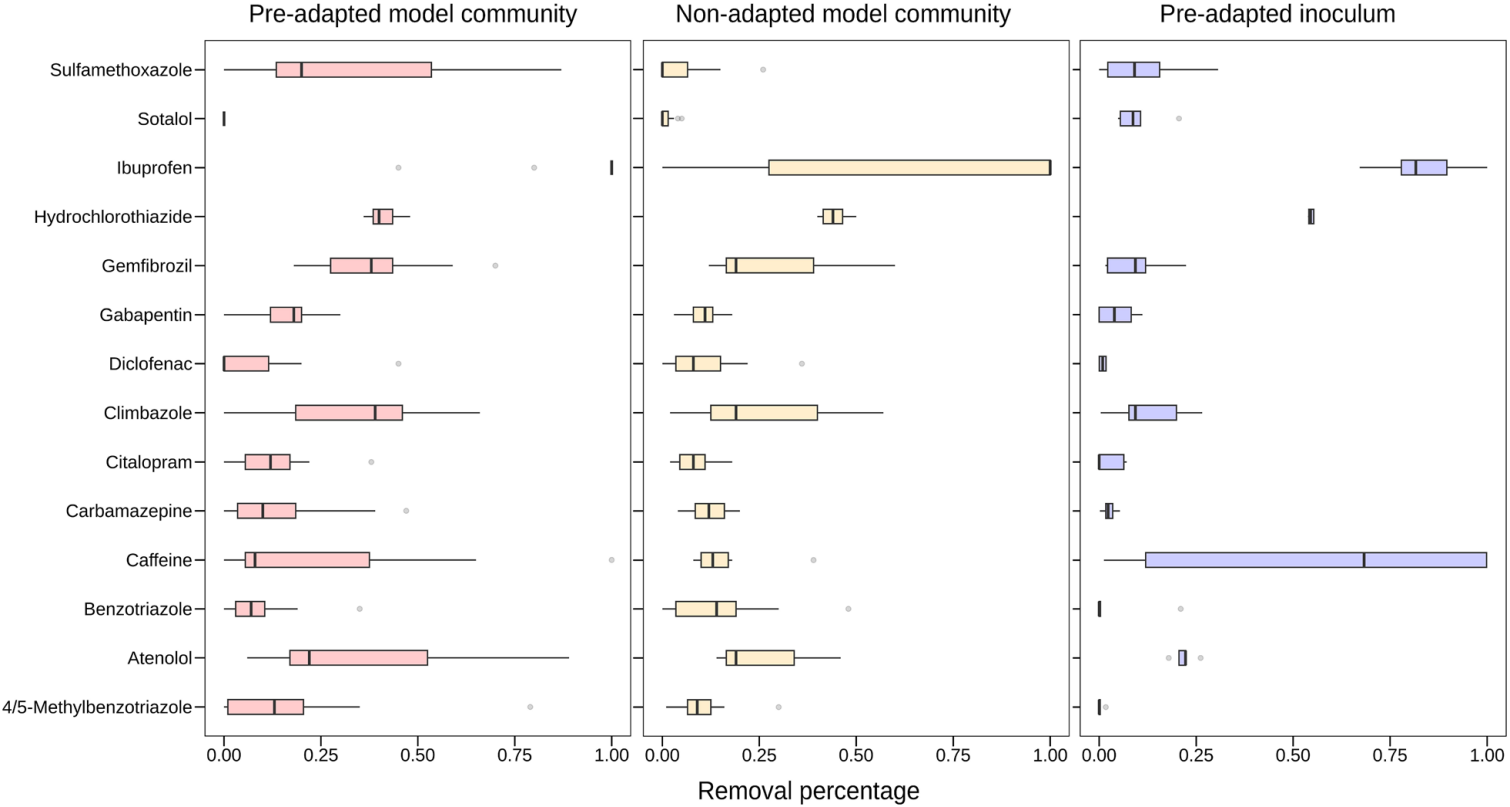
TOrC removal by 15 model communities derived from pre-adapted inoculum (left), 15 model communities from non-adapted inoculum (middle), and 5 pre-adapted inocula (right). TOrCs with below 20% removal in all communities were excluded

Overall, the thirty selected model communities in stage 3 were able to transform 17 of the 27 TOrCs. Ten residual TOrCs remained unchanged within the microbial model communities. Most of the communities could effectively degrade three to six TOrCs simultaneously (range: 1–9; 20% cutoff of removal rate), but to different degrees (Additional file 2: Fig. S6). Moreover, only model communities from pre-adapted communities exhibited transformation for more than six compounds. Specifically, the average removal of 17 TOrCs by 15 communities from the pre-adaptation group was 30.1%, and the percentage in the non-adaptation group was 22.4% (*t*-test, p = 0.16). There were more TOrCs degraded after pre-adaptation (n = 17), including some persistent compounds such as carbamazepine (46.5%) and gabapentin (25.2%), which were removed below 20% in the communities from the non-adaptation group (n = 10) (Fig. 6). Comparing the removal pattern of pre-adapted consortia and the subsequent model communities, we could observe that some model communities had similar removal on for example hydrochlorothiazide, ibuprofen and caffeine, some had higher removal than the whole community on sulfamethozaxole, gemfibrozil, climbazole and atenolol. There were even unchanged compounds by pre-adapted inocula that exhibited degradation by model communities (i.e., carbamazepine, diclofenac, gabapentin, citalopram, 4/5-methylbenzotriazole). The reduction of these 17 TOrCs was attributed to biodegradation as there was almost no abiotic degradation indicating by controls (< 3.5%).

### Relationship between TOrC removal performance and microbial traits

**Fig. 7:**
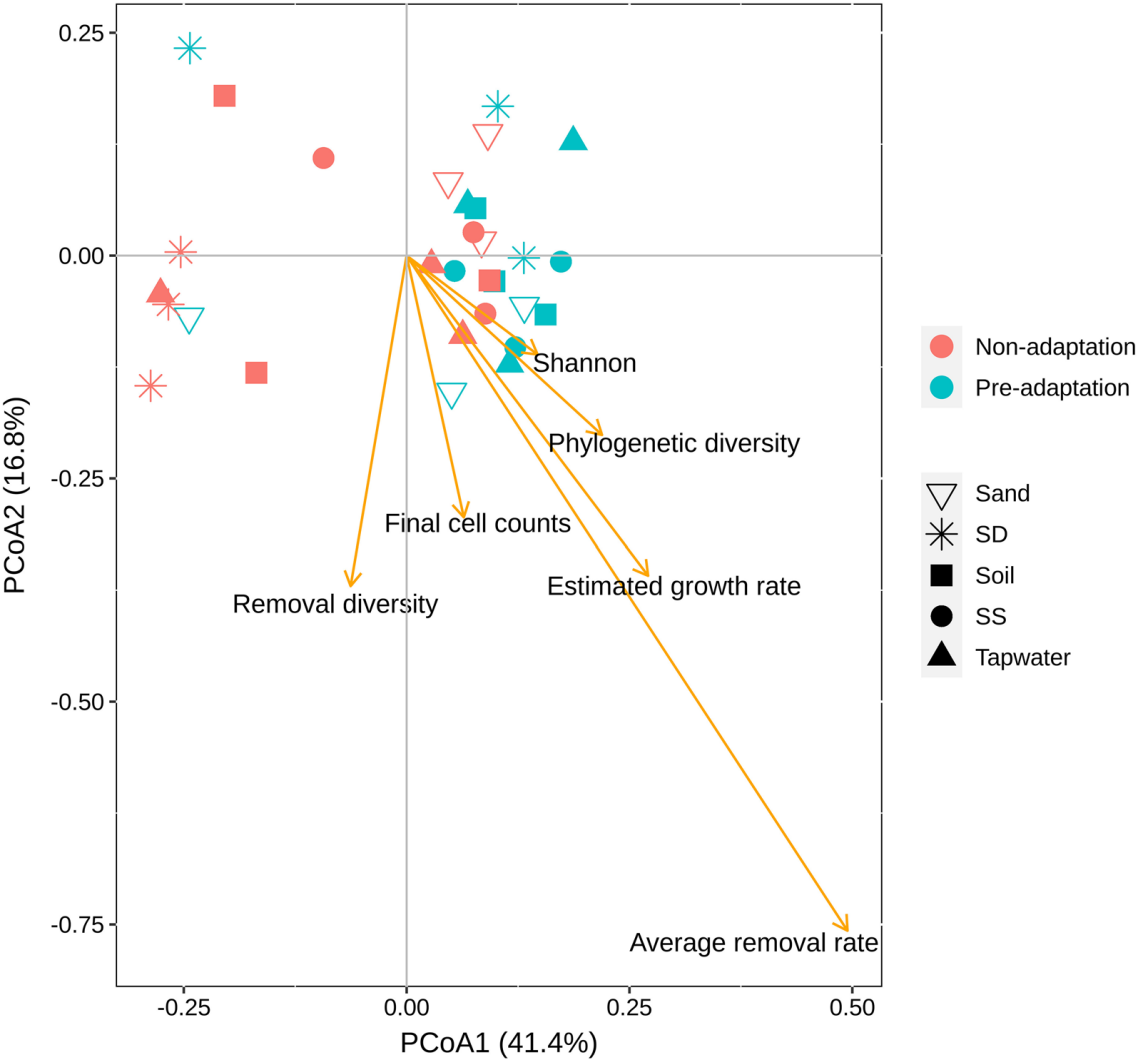
Principal coordinate analysis (PCoA) of TOrCs removal by thirty model communities based on Euclidean dissimilarities. The environmental variables determined using envfit function in vegan were displayed as vectors, with a length proportional to the correlation between the variable and the PCoA ordination

The relationships between 17 TOrC removal performance and potential microbial traits were investigated by principal coordinate analysis (PCoA) across microbial communities in the pre- and non-adaptation groups. The two main axes explained 41.4% and 16.8% of the variance, respectively (Fig. 7). A weak separation was observed between pre- and non-adaptation groups (Adonis, R^2^ = 0.095, p = 0.019) regarding their TOrC removal patterns. We hypothesized that phylogenetic diversity of model communities will influence TOrC removal rates, which could explain the dissimilarity between groups. However, the envfit analysis showed that the variation was not correlated with phylogenetic diversity (Adonis, R^2^ = 0.089, p = 0.3). We also tested the correlation with other variables, i.e., Shannon diversity, estimated growth rate, final cell counts (cell counts at d21), 27 TOrCs average removal rate and removal diversity (number of TOrCs above 20% simultaneous removal). We found that only average removal rate (Adonis, R^2^ = 0.82, p = 0.001) and estimated growth rate (Adonis, R^2^ = 0.20, p = 0.046) exhibited significant correlation with the variance, indicating the positive effect of model communities’ growth rates on the overall TOrC removal performance. Although phylogenetic diversity was not related to TOrC removal rates, it was found to have a positive correlation with removed TOrC numbers (cor.test, R^2^ = 0.39, p = 0.03).

## Discussion

In the absence of mechanisms of TOrC biotransformation by microbiome in the aquatic systems, it is often difficult to develop high-efficiency TOrC-specific biological treatment technologies in the engineering field. Deciphering the complexity of microbial functions can be achieved by starting with simplified systems, which relies on controllable bottom-up experiments with a few species [46]. In this study, by growing serially diluted tap water, sediment, sand, and soil under oligotrophic condition with TOrCs as the sole carbon source to generate self-assembled model communities, we investigated how the pre-adaptation of inoculum could impact the diversity of model communities and their removal on TOrCs. We found that while pre-adaptation process reduced the overall richness and diversity of inoculum, it resulted in greater diversity of model communities that can survive or degrade TOrCs. Pre-adaptation also enhanced TOrC removal performance in terms of overall removal rates and degradable TOrC numbers. Our hypothesis of phylogentic diversity’s influences on TOrC removal rates was rejected, as no significant correlation was identified. However, higher phylogenetic diversity in terms of e.g. phyla of model communities will require further investigations and could lead to more removed TOrCs.

### Advantages of the model community establishment workflow

There have been a variety of microbial model communities developed for different purposes by different approaches. They can be mutant-based communities, the multispecies synthetic communities and the (semi-) natural communities as Bengtsson-Palme [14] suggested. Compared with those reported model communities [47–49], our workflow (Fig. 1) has the strong advantages of, firstly, high-throughput cultivation under highly controlled and well-understood conditions which allows large numbers of varying diversity communities providing a more reliable reflection of natural microbial ecosystems. We started with 4707 diluted communities in stage 2 and 2658 of them showed successful growth. In addition to our selection of 95 communities and resulting 30 taxonomically non-redundant ones, it is possible to enlarge the scale with changeable inoculum and synthetic media for different purposes [18, 20, 50]. Secondly, addressing the defects of conventional isolation and artificial assembly, our method has the potential to study uncultured microorganisms or strains that cannot survive individually, as well as the rare species which usually account for less than 5% of the community but can contribute disproportionately to the microbial functions [51]. For example, *Micrococcaceae* dominant in model communities were diluted from the non-adapted soil inoculum (Fig. 5), whereas it is only present as 0.3% in the initial soil communities.

### Standardization effect of pre-adaptation on the microbial diversity

Pre-adaptation has been proven to be a key step in our method. It serves as a selective enrichment and a standardization process reducing and normalizing microbial diversity of varying inocula and further facilitating the species distribution in model communities. It is widely accepted that assessing the diversity of different microbes requires standardization [52], similarly, to establish model communities from various natural microbial systems via a common workflow, standardized initial samples are necessary. The pre-adaptation step offered an opportunity to scale down the species richness of varying communities to similar ranges and of the same dilution thresholds (e.g., 10 cells/mL), which made the subsequent model communities more comparable and adaptable for many different approaches (Fig. 2). In our study, the species richness and phylogenetic diversity of pre-adapted consortia decreased significantly, indicating an initial filtering effect of pre-adaptation resulted in microbial structure shifts and biodiversity loss in response to environmental stress [53]. The most noticeable change was the 59% to 8% reduction on rare taxa (≤ 2% abundance) (Additional file 2: Fig. S4), which appeared to be more sensitive to environmental pressure (in our case is increasing concentrations of TOrCs) than abundant species. The sensitivity of rare taxa is also supported by other studies [54–56], for example, Yi et al. [57] found that abundant microbes established cooperative interactions and competed for resources and ecological niches with rare species under the stress of benzo[a]pyrene. Different explanations have been proposed that rare taxa have the ability to become dominant in the community and with the increased abundance, they could have higher functional importance than the other abundant species, the so called “insurance effect” help microbial systems maintain their functions under environmental changes [54, 58]. This phenomenon was also observed in our experiments that *Nocardiaceae* developed from rarity to dominance after pre-adaptation (Additional file 2: Fig. S5). Interestingly, the loss of biodiversity in pre-adapted inocula did not lead to low diversity of model communities, in contrast, the species distribution evenness and phylogenetic diversity were notably higher in model communities derived from pre-adaptation than that from non-adaptation (Fig. 4). One explanation could be the TOrC stress acting as an environmental filter during the pre-adaptation process induced stable TOrC-degrader communities (specialists), while the non-adapted inoculum contained mostly taxa adapted to other environmental niches or generalists. Therefore, when we diluted the inoculum, with the reduction of microbial populations specialists had higher chances to co-exist due to their cooperation effect and maintain the functional stability [59]. However, the non-adapted microbes could compete for resources and niches, thus leading to diversity loss in model communities when facing sudden environmental fluctuations (similar with the non-adaptation to pre-adaptation trend). The microbial responses to TOrCs could be further studied at higher temporal resolution within the pre-adaptation period in terms of compositional and functional changes.

### Phylogenetic diversity and microbial growth rates facilitated TOrC removal

Pre-adaptation also has positive effects on TOrC removal performance in terms of removal rates and degradable TOrC number. The necessity of adaptation ranging from several months to years of microbial communities for removing trace pollutants has been suggested previously [34, 60–62]. In these studies, some reported microbial adaptation resulted in the enhancement of TOrC degradation, whereas opposite results were found that pre-adaptation did not affect their attenuation. Our findings supported the former, in general, the overall 17 TOrCs removal rates were increased by pre-adapted model communities (30.1% vs. 22.4%). In addition, there were more TOrCs that could be transformed by adapted model communities (n = 17) than non-adapted model communities (n = 10). The other ten unchanged TOrCs in our experiments i.e., amisulpride, antipyrine, candesartan, fluconazole, primidone, sotalol, tramadol, trimethoprim, venlafaxin, 4-formylaminoantipyrine, have been reported as persistent compounds with very low removal in biological treatment (Additional file 1: Table S1). Interestingly, the TOrC removal diversity rather than the removal rates was found to be positively related to phylogenetic diversity of model communities.

Microbial diversity is considered to be essential for facilitating ecosystem functions via niche partitioning effects and interaction effects [63, 64]. Although we did not identify the diversity enhancement on overall TOrC removal rates, which is still in accordance with other studies [65, 66], the benefits from phylogenetic diversity were revealed by more degradable TOrCs. A possible explanation of this could be that the niche space overlap of more distantly related species is expected to be less than closer species, thus potentially favoring niche expansion to utilize more resources [26]. This niche expansion is even stronger when partners are metabolic specialists rather than generalists, and it allows the pairing of auxotrophic taxa with metabolic dependencies that could add additional functional genes [67]. For example, one of our model communities harbored a *Phenylobacterium* (Additional file 2: Fig. S4), which is the single described species lacks the vitamin B_12_ pathway and requires a B_12_ producer in its community, but potentially adds pathways related to chloridazon, antipyrin and pyramidon degradation [68]. As we discussed above, pre-adaptation could facilitate specialists for TOrC degradation, therefore, model communities have more possibilities to transform a wider range of compounds. In our case, pre-adapted model communities growing in the medium containing 27 mixed TOrCs can only remove nine compounds above 20%. This maximum number may be limited by their diauxic growth pattern (i.e., TOrCs are consumed sequentially or selectively) rather than co-utilization when faced with multiple carbon sources, especially when the carbons sources are toxic and refractory chemicals [69, 70]. The variance of TOrC removal rates between pre- and non-adaptation groups was found to be related to the estimated growth rate, indicating that the faster growth of model communities could predict the better removal of TOrCs. This could also be supported by the diauxic growth as the order of substrates consumption is determined by the biomass and growth rate when the same compounds are served as sole carbon sources [71]. More researches can be done by monitoring the TOrC biotransformation kinetics (in stage 3) which better indicates the relationship between cell growth and TOrC removal.

To our best knowledge, this is the first study that investigated a wide range of TOrC biotransformation based on different model communities derived from varying natural microbial systems. Although there have been previous attempts addressing TOrC biotransformation mechanisms [18, 49], they have limitations that either the systems themselves are too complex (e.g., soil and sludge) to characterize the key players and microbial functions, or too simplified (e.g., isolates degrading specific compound) to be applicable under environment conditions. Our method with reduced richness model communities serves as a compromise, which scales down the complex microbial interactions but their diverse combinations are still reflective of the actual environmental communities. This robust and standardized protocol can also provide a basis for studies interested in specific or diverse TOrCs (or other pollutants) biotransformation, as the inoculum and the identity of the chemical filters (in our case, the 27 TOrCs) are exchangeable. In future research, we can use these model communities to identify key driving agents of biotransformation (i.e., relevant microbes and their interactions, metabolic pathways down to the enzyme/gene level, and roles of co-substrates and cofactors) at a well-defined and standardized community level.

## Conclusion

Complete understanding of TOrC biotransformation is essential for the development of biological treatment methods in aquatic environment. This study set up a robust and standardized workflow for establishing low complexity model communities to investigate TOrC biotransformation mechanisms driven by interacting taxa. Our experimental results demonstrated that the pre-adaptation of natural communities to TOrC environment reduced and standardized the diversity of varying inocula. In contrast, the pre-adaptation step improved the diversity of resulting model communities in terms of species distribution evenness and phylogenetic diversity as well as the average TOrC removal rates. The phylogenetic diversity was further found to be positively related to number of TOrCs that can be biodegraded simultaneously. However, the average TOrC removal was not well correlated to the observed changes in phylogenetic diversity but to the growth rates of model communities.

### Supplementary Information

**Additional file 1: Table S1.** The names, structure, uses, occurrence, RQ values and biotransformation efficiencies of 27 TOrCs used in this study.

**Additional file 2: Figure S1.** Cell counts after 21 days incubation in six stages. **Figure S2.** Cell counts of communities growing from different diluted cell numbers (below growth threshold) in the (a) pre-adaptation and (b) non-adaptation group after 21 days incubation, n = 48. **Figure S3.** Heatmap illustrating 27 TOrCs removal efficiencies by thirty microbial communities. The color legend represents the removal percentage.** Figure S4.** Taxonomic composition of 11 model communities selected after TOrC removal performance assessment at the genus level. Numbers in the pie chart represent the OTUs belonging to each genus. **Figure S5.** Comparison of microbial structure between pre- and non-adapted inocula at the family level. **Figure S6.** Thirty model communities’ frequency on simultaneously removed TOrC number. The removal cutoff is 20%.

**Additional file 3.** OTU tables and metadata: otu_tab_95_model_communities.csv, otu_tab_inocula.csv, metadata.csv.

## Data Availability

Amplicon sequencing data has been deposited at INSDC (with ENA: https://www.ebi.ac.uk/ena) under the project accession number PRJEB63566. The OTU tables are deposited as csv files (see Additional file 3).
