# Supplementary material for: Establishment of microbial model communities capable of removing trace organic chemicals for biotransformation mechanisms research
Source: Microb Cell Fact. 2023 Dec 2;22:245. doi: 10.1186/s12934-023-02252-6 (PMC10693053; doi:10.1186/s12934-023-02252-6)
Supplement: Supplementary file 1 — Additional file 1: Table S1. The names, structure, uses, occurrence, RQ values and biotransformation efficiencies of 27 TOrCs used in this study. [file 12934_2023_2252_MOESM1_ESM.docx]

Establishment of microbial model communities capable of removing trace organic chemicals for biotransformation mechanisms research

(Supplementary Material)

Table S1. The names, structure, uses, occurrence, RQ values and biotransformation efficiencies of 27 TOrCs used in this study.

| **Compound** | **Structure** | **Use** | Occurrence (μg/L) | RQ | **Biotransformation efficiency (%)** | **Reference** |
| --- | --- | --- | --- | --- | --- | --- |
| Acesulfame | 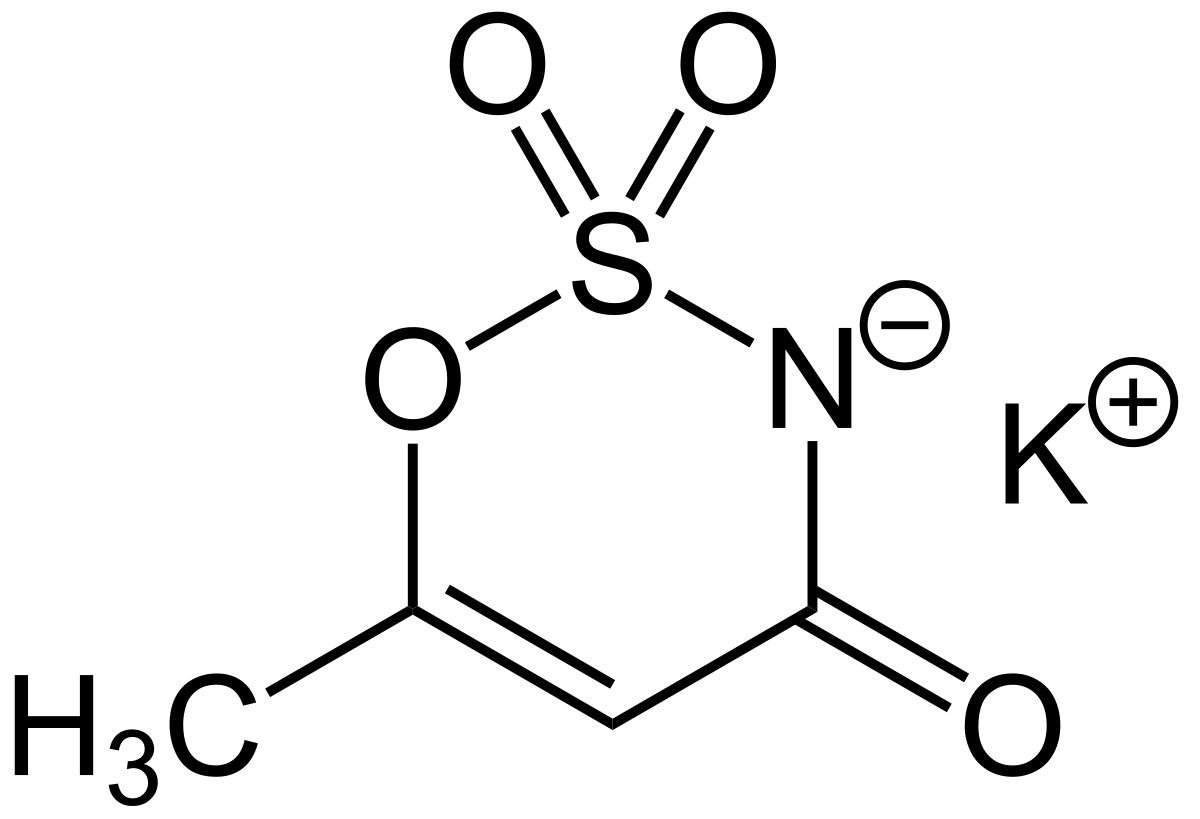 | Sweetener | ND-46 | 0.05 | 85 | Buerge *et al*. 2009;  Kahl *et al*. 2018;  Huang *et al*. 2021;  Shen *et al*. 2023 |
| Amisulpride | 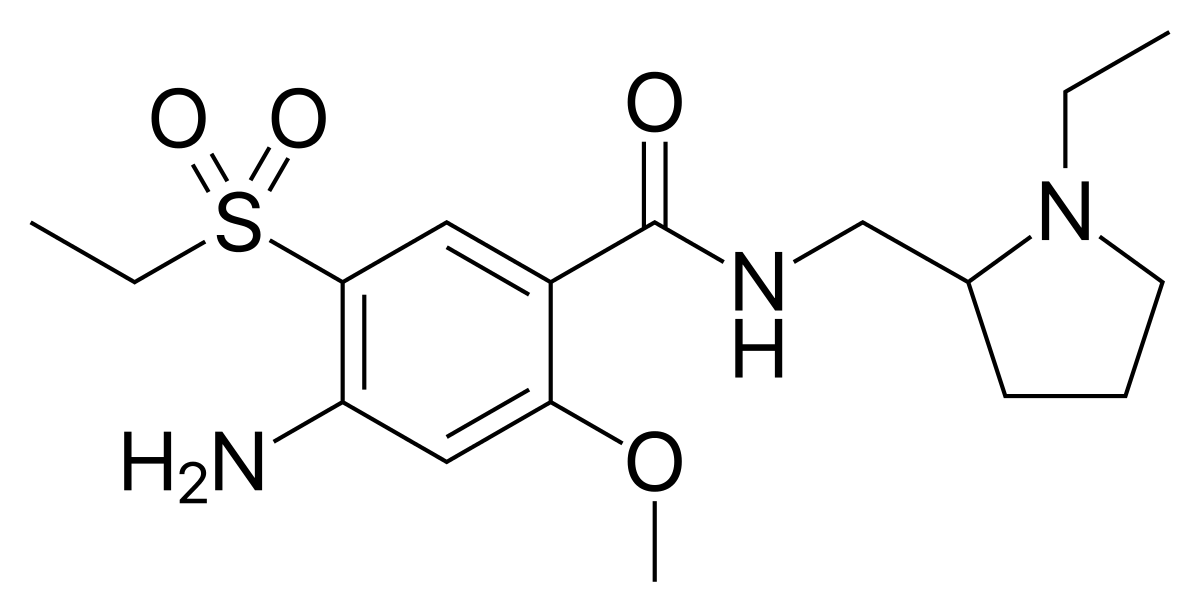 | Neuroleptics | ND-1.32 | 0.1-1 | 0 | Bollmann *et al*. 2016;  Athanasakoglou *et al*. 2021;  Kucharski *et al*. 2022 |
| Antipyrine | 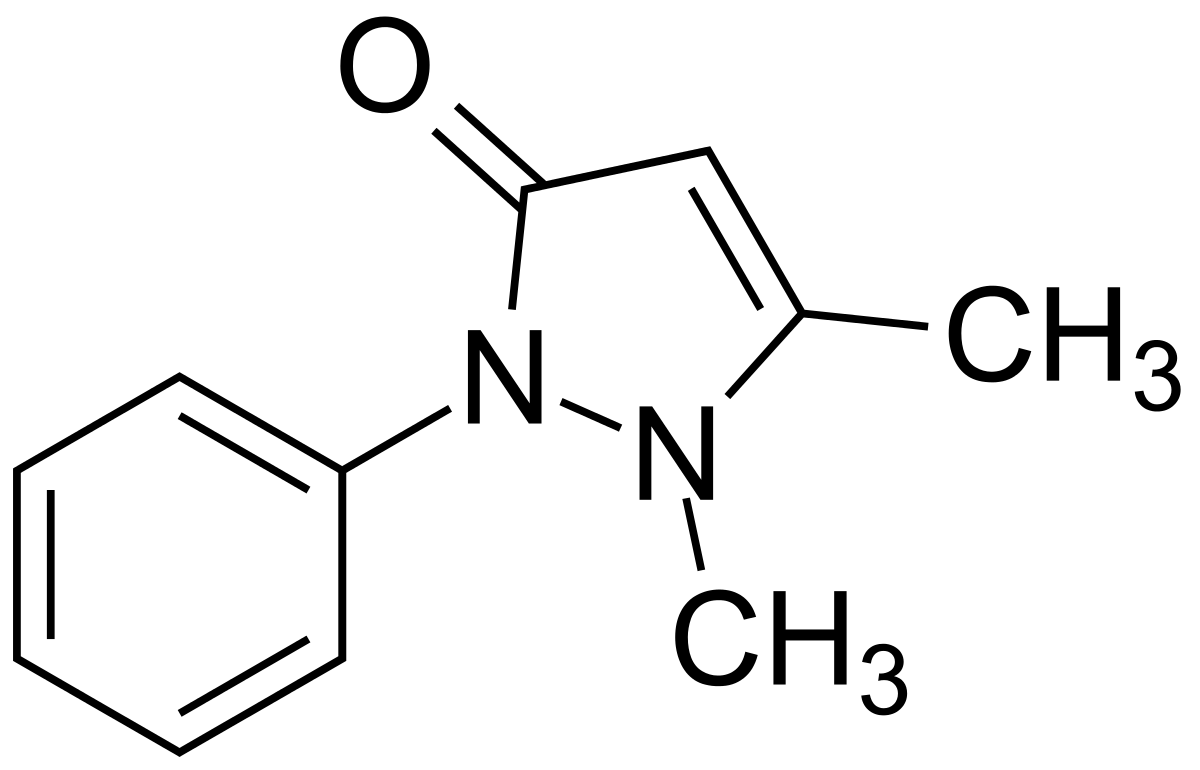 | Analgesic | 1.302 | 9.98 × 10^-6^ | 6.5 | Gimeno *et al*. 2016;  Rapp-Wright *et al*. 2023;  Kim *et al*. 2023 |
| Atenolol | 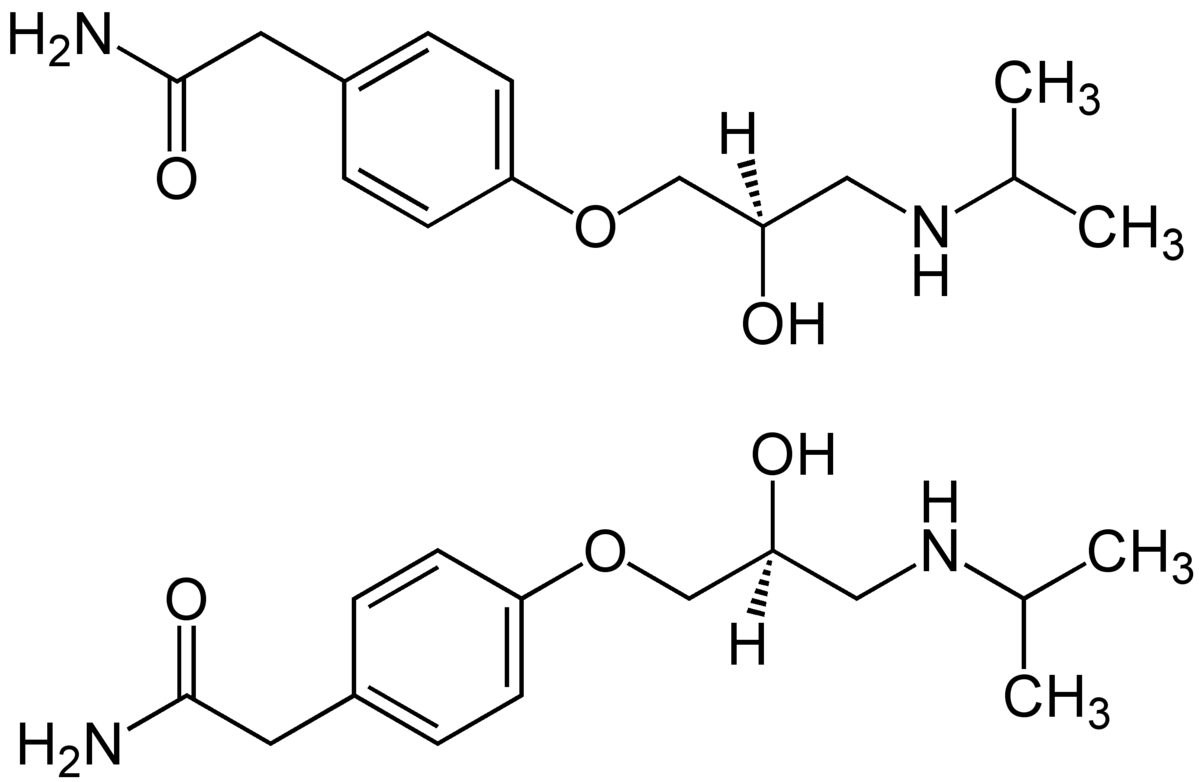 | Beta-Blockers | ND-26.5 | 0-0.014 | <0-85.1 | Luo *et al*. 2014;  Khasawneh *et al*. 2021;  Yi *et al*. 2022 |
| Benzotriazole | 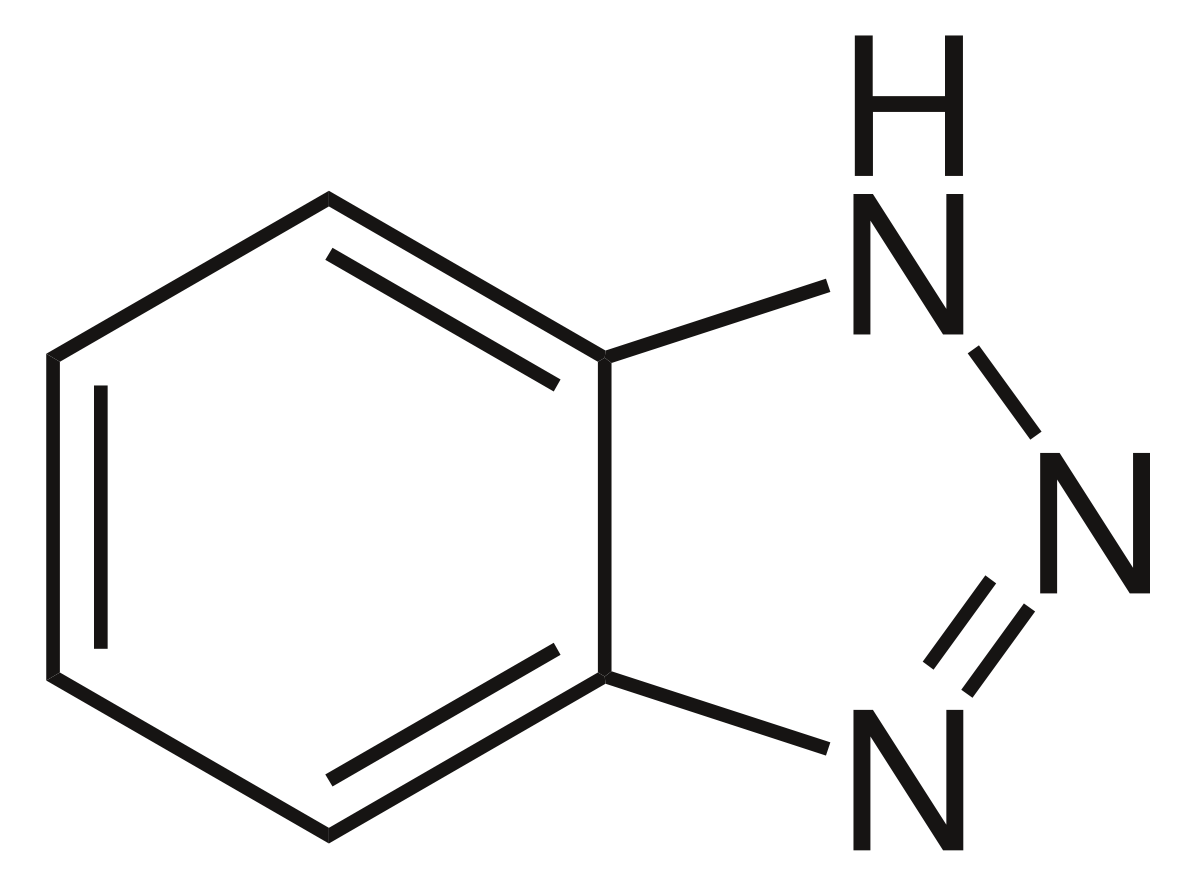 | Industrial | ND-100 | 0-0.011 | 40-70 | Alotaibi *et al*. 2015;  Torresi *et al*. 2019;  Khare *et al*. 2023 |
| Caffeine | 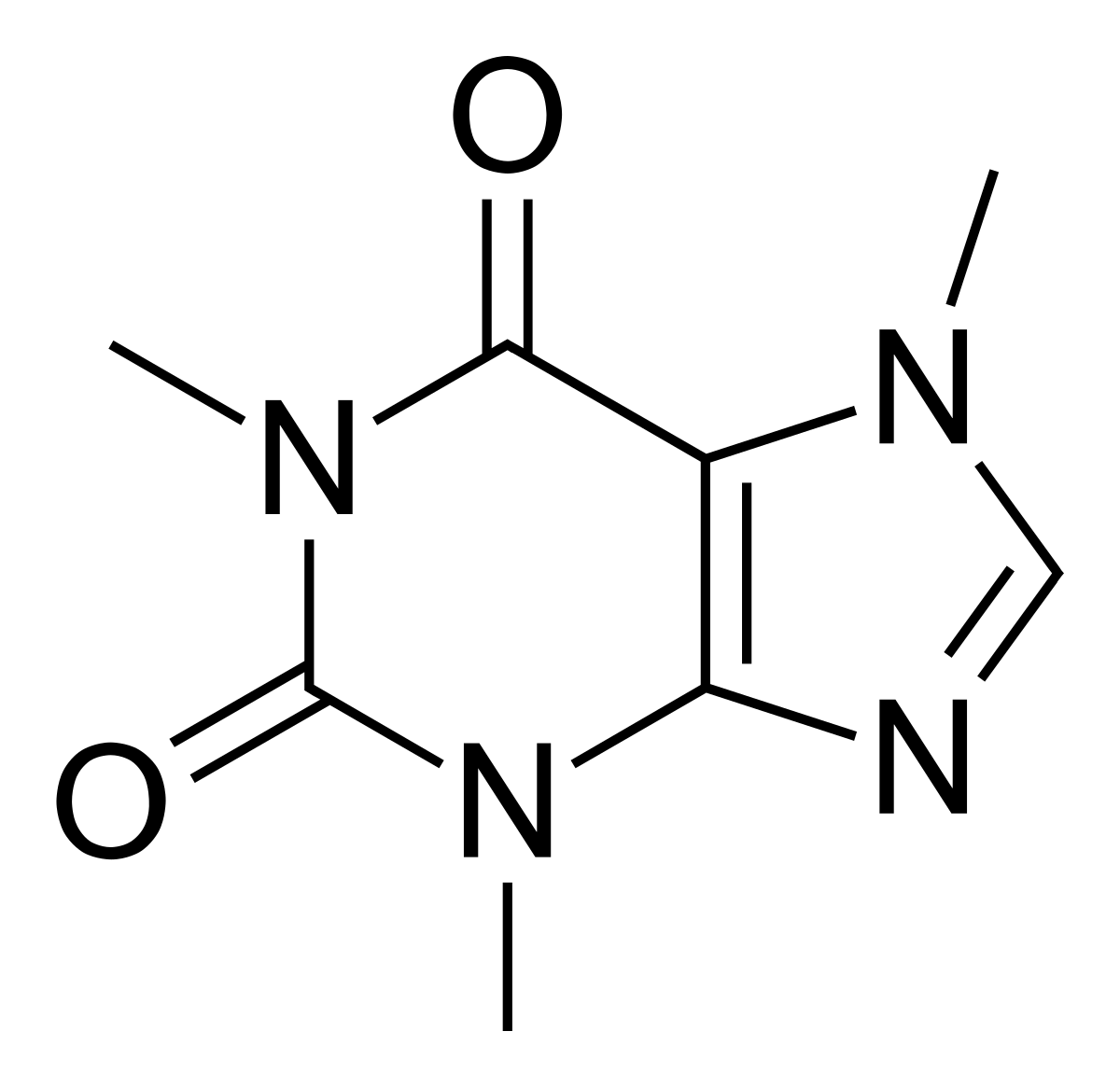 | Psychoactive drug | ND-220 | 0.02 | 49.9-99.6 | Luo *et al*. 2014;  Khasawneh *et al*. 2021;  Zhou *et al*. 2022 |
| Candesartan | 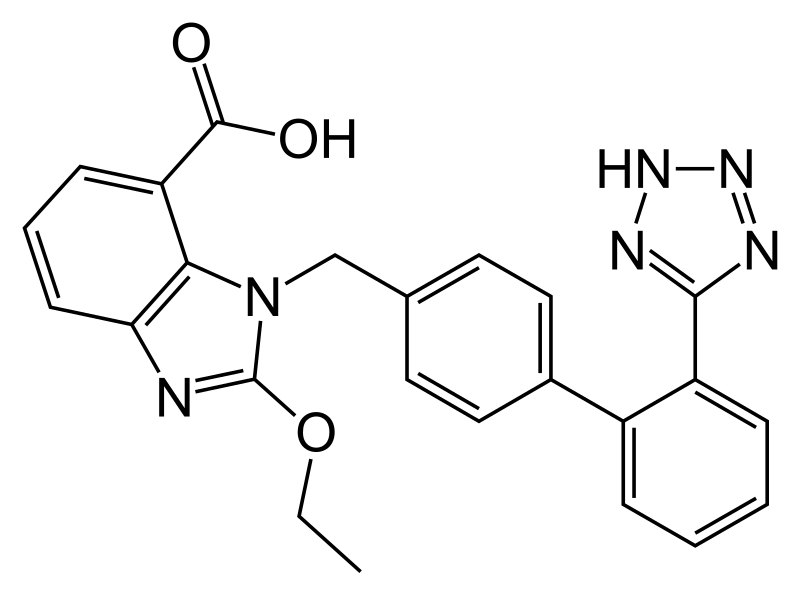 | Angiotensin receptor blocker | ND-1 | 0.009 | <20 | Bayer *et al*. 2014;  Burke *et al*. 2018 |
| Carbamazepine | 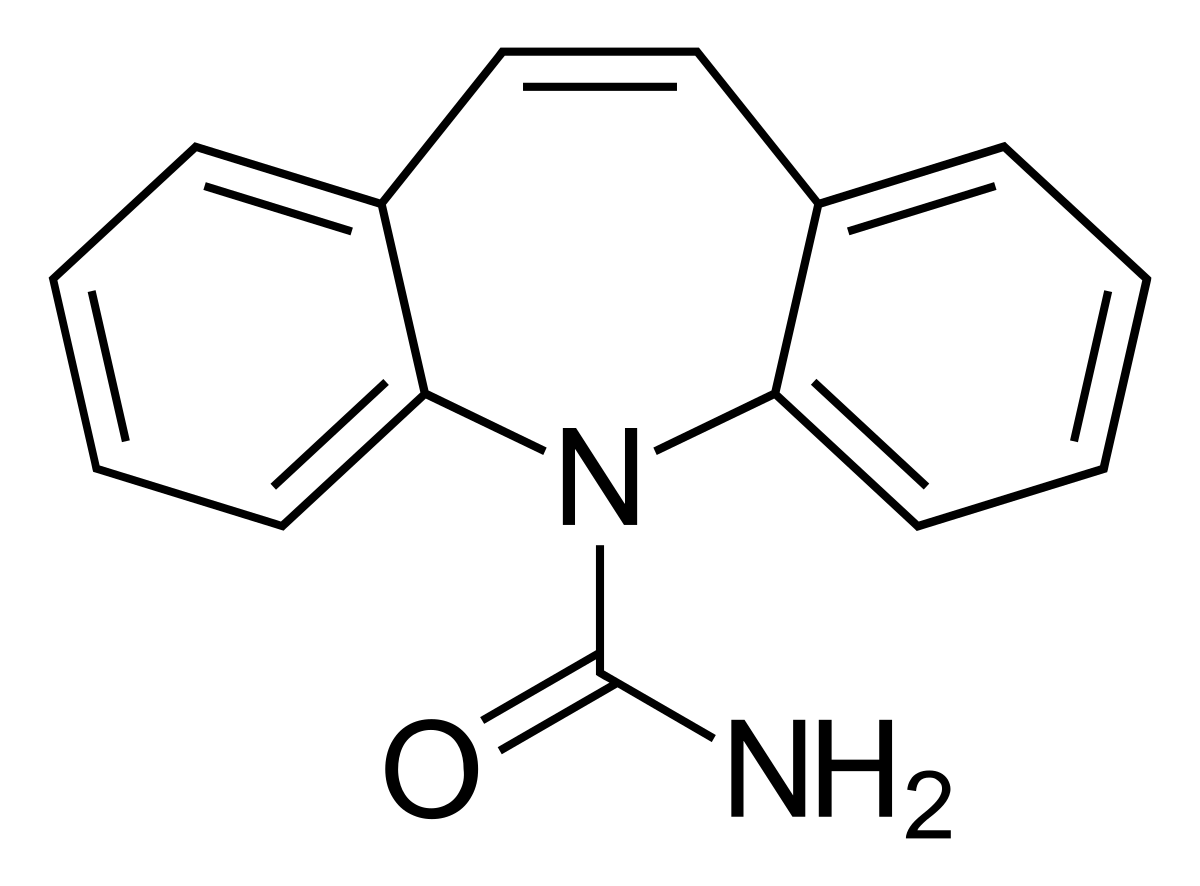 | Antiepileptics | ND-38.24 | 0-1.8 | <0-62.3 | Luo *et al*. 2014;  Khasawneh *et al*. 2021 |
| Citalopram | 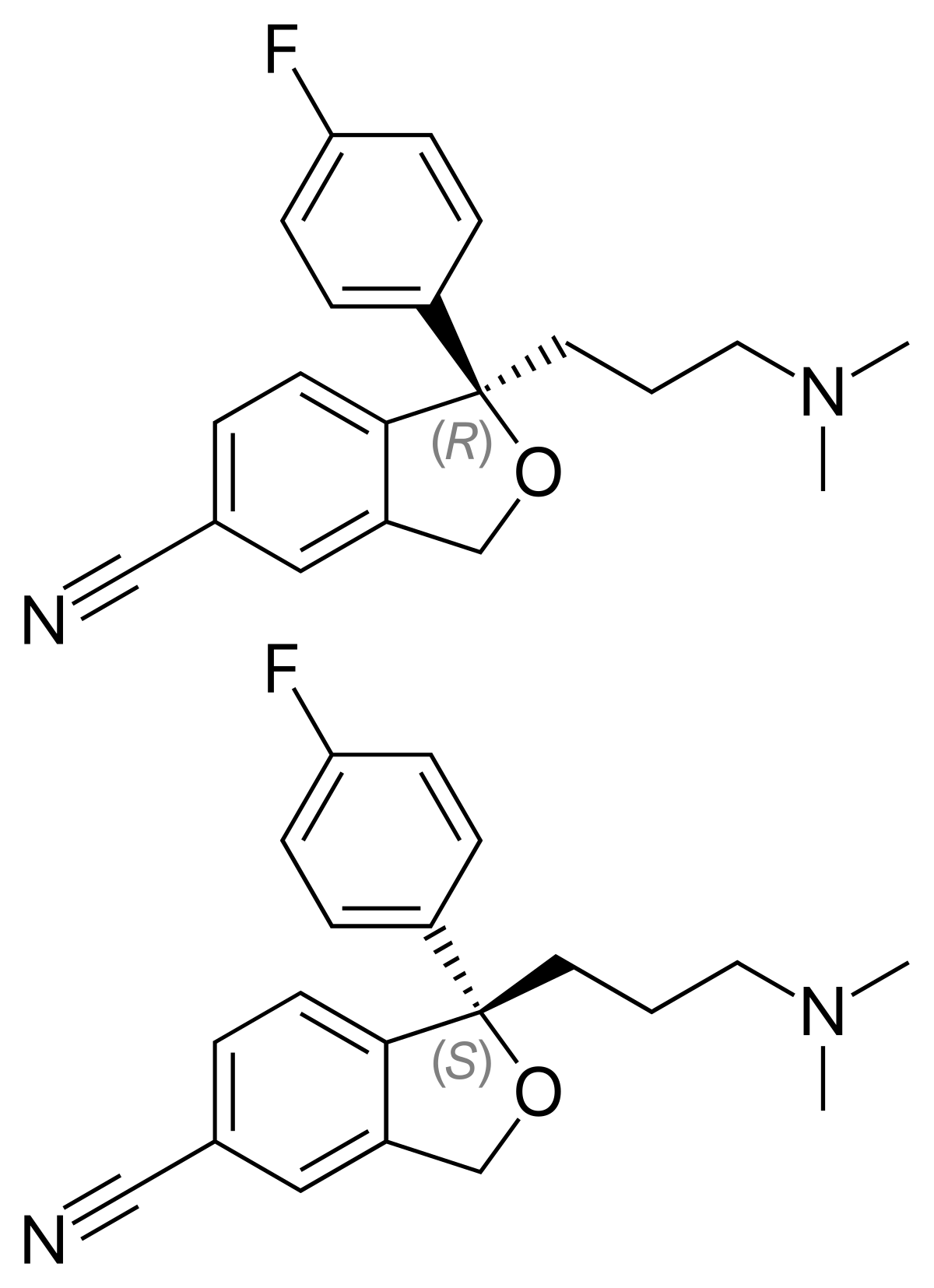 | Antidepressant | ND-84 | 1.55 | 40-60 | Cunha *et al*. 2017;  Suarez *et al*. 2010;  Singh *et al*. 2022 |
| Climbazole | 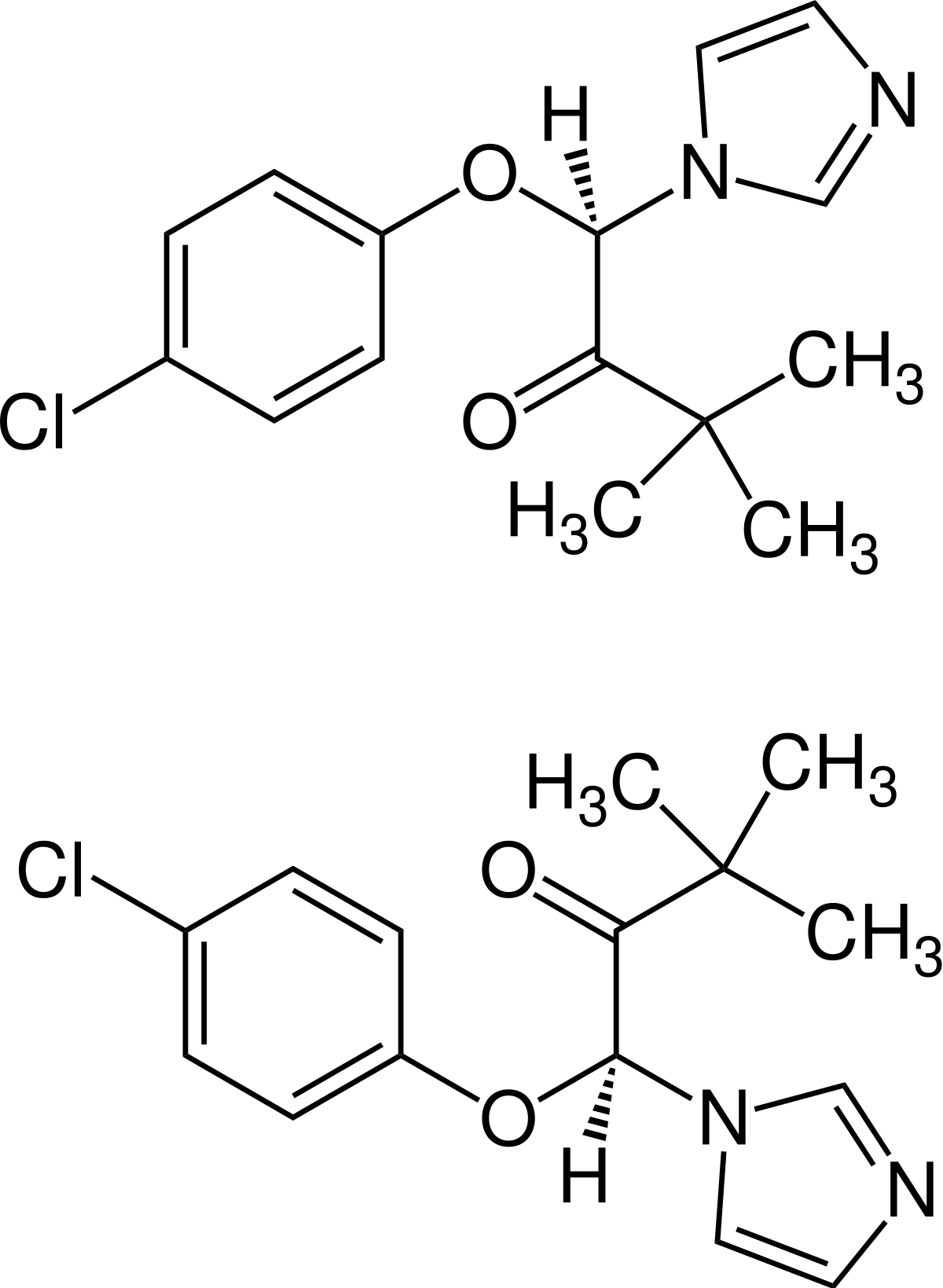 | Antifungal | ND-0.465 | 0.007-0.035 | 88 | Pan *et al*. 2018;  Selak *et al*. 2022;  Anagnostopoulou *et al*. 2023 |
| Diclofenac | 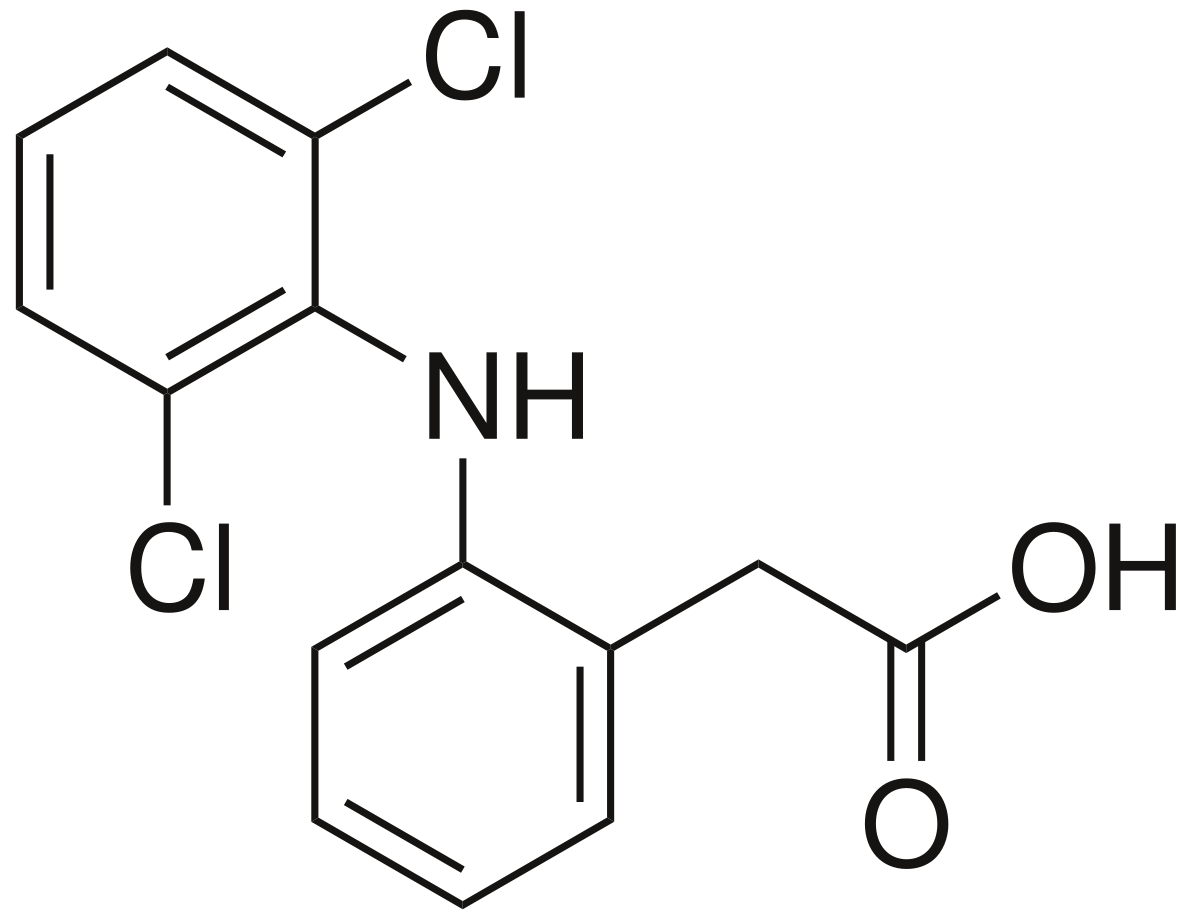 | Antiinflammatory | ND-22.8 | 0-43.6 | <0-81.4 | Luo *et al*. 2014;  Zur *et al*. 2020;  Khasawneh *et al*. 2021 |
| Fluconazole | 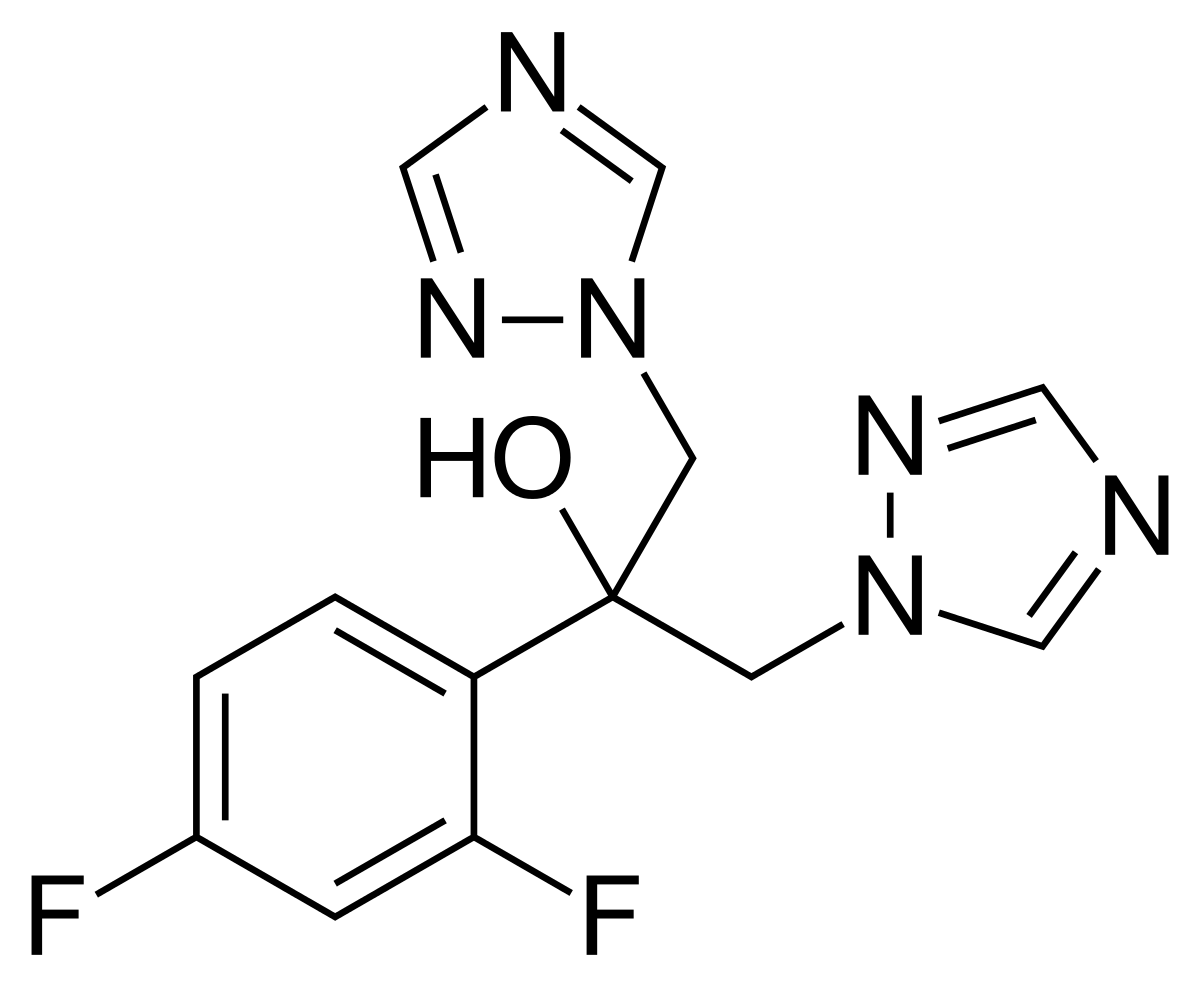 | Antimycotics | ND-13.2 | 0.0002-0.11 | 1 | Faria *et al*. 2020;  Assress*et al*. 2020;  Monapathi *et al*. 2021 |
| Gabapentin | 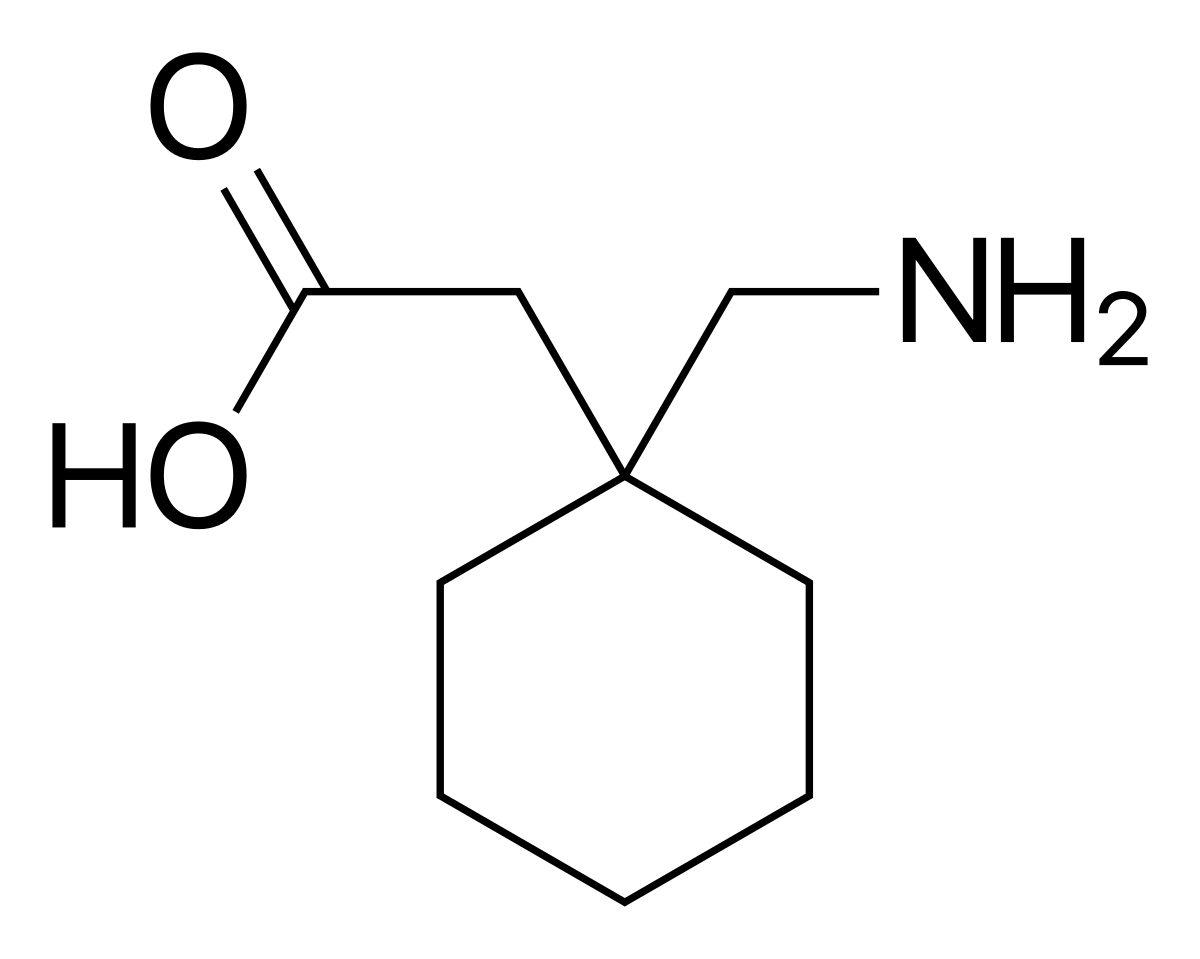 | Anticonvulsant drug | 0.79-15.36 | 1.32 | 7.9 | Herrmann *et al*. 2015;  Daouk *et al*. 2016;  Khasawneh *et al*. 2021 |
| Gemfibrozil | 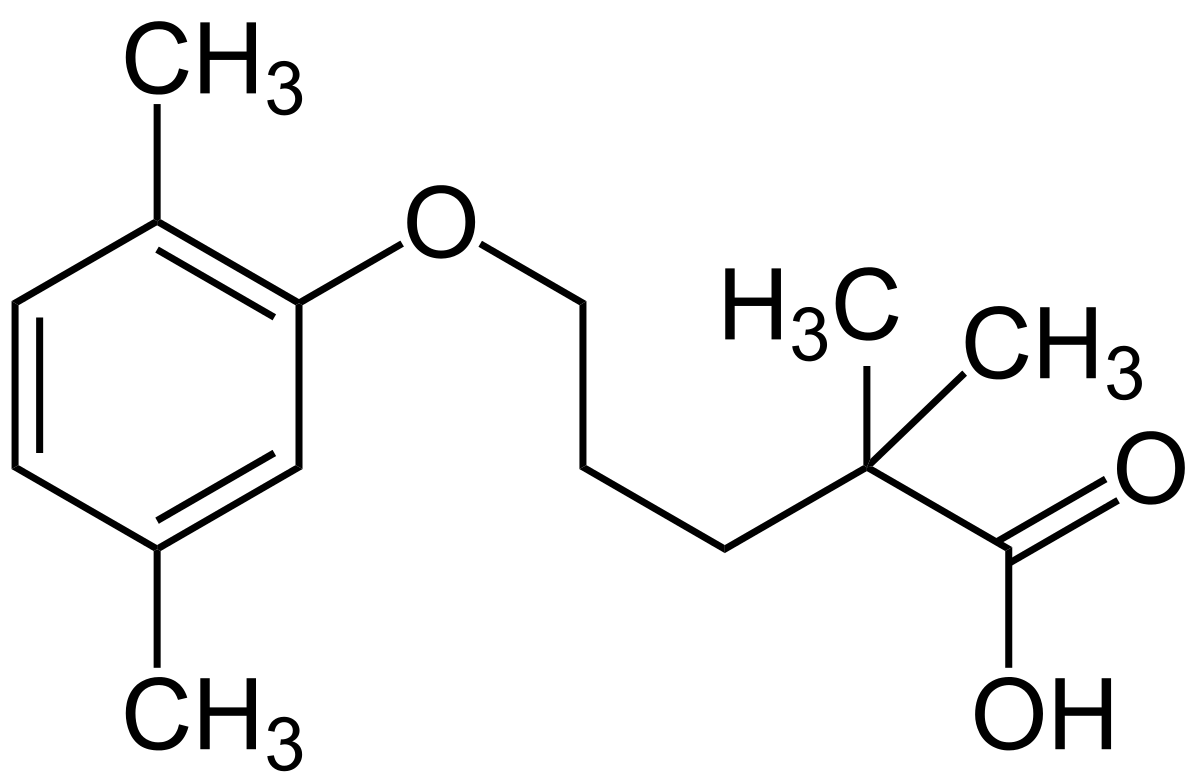 | Lipid-lowering drug | ND-76.1 | 0.019-22.3 | <0-92.3 | Luo *et al*. 2014;  Kjeldal *et al*. 2016;  Khasawneh *et al*. 2021 |
| Hydrochlorothiazide | 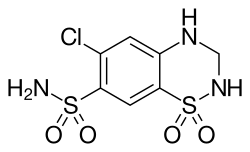 | Diuretics | 1 | 0.01-0.1 | 38 | Sipma *et al*. 2010;  Chen *et al*. 2016;  Díaz-Garduño *et al*. 2017 |
| Ibuprofen | 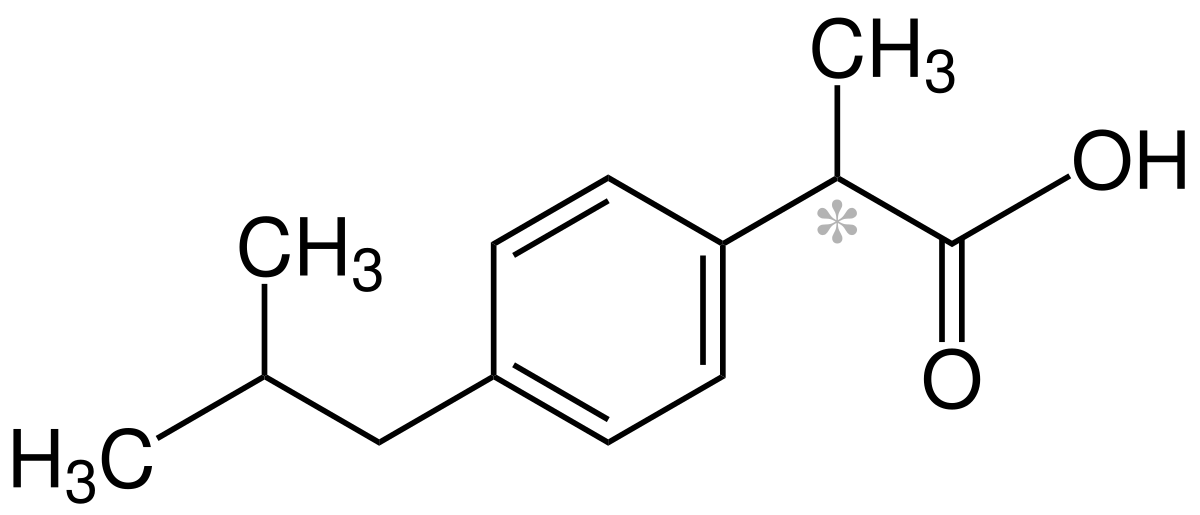 | Antirheumatic drug | ND-303.0 | 0-5.42 | 72-100 | Luo *et al*. 2014;  Almeida *et al*. 2013;  Khasawneh *et al*. 2021 |
| Iopromide | 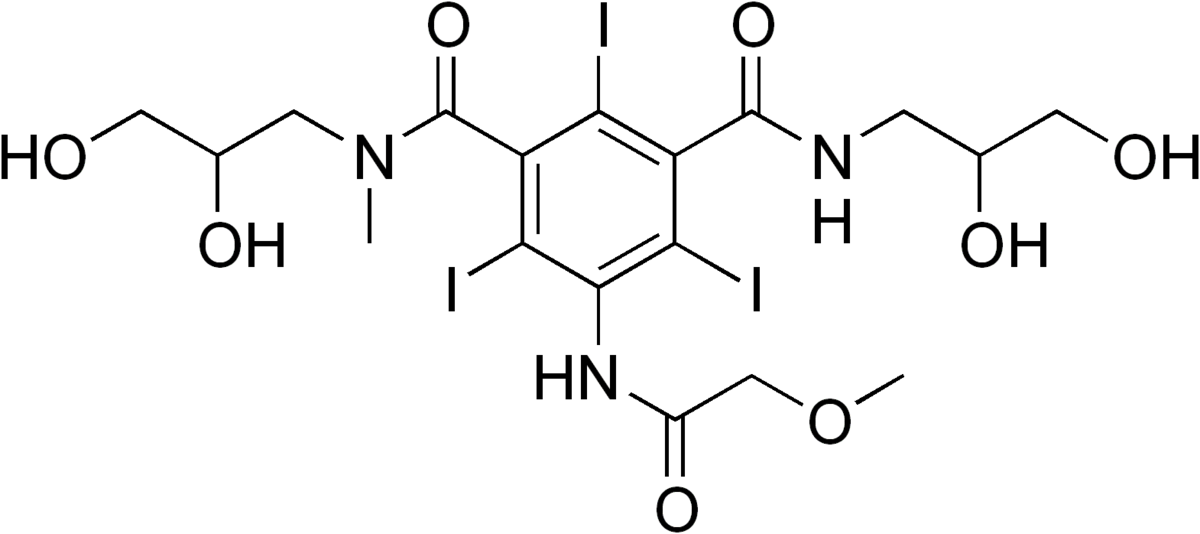 | Contrast media | 0.02-1400 | 0.1-1 | 51 | Sipma *et al*. 2010;  Schulz *et al*. 2008;  Kim *et al*. 2007;  Al Aukidy *et al*. 2014 |
| Metoprolol | 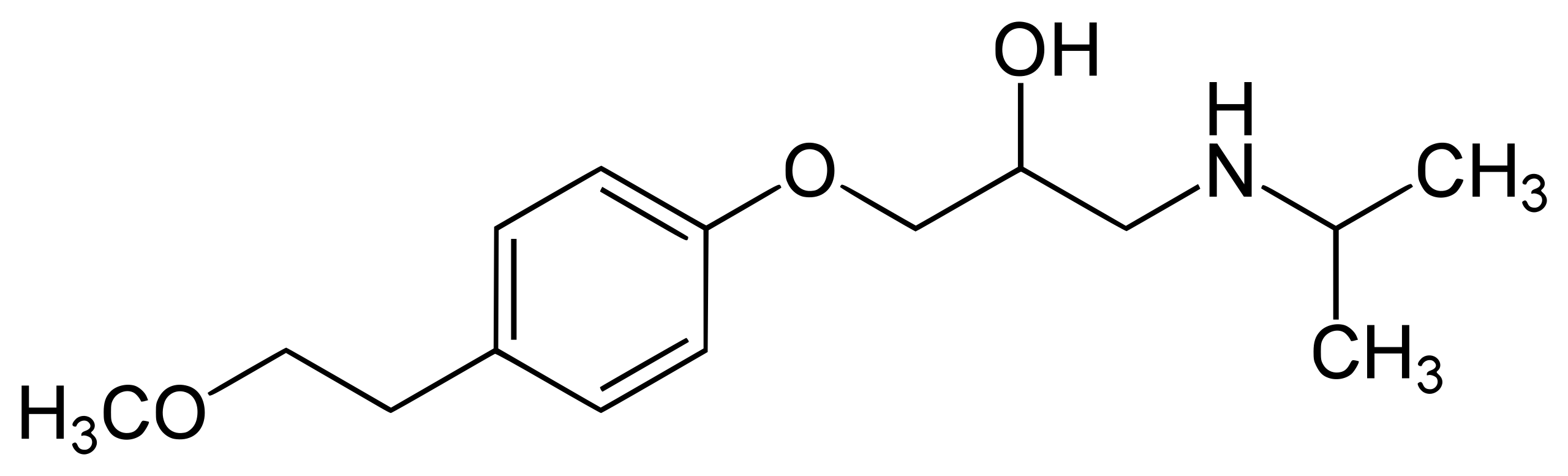 | Beta blockers | ND-5.76 | 0.02 | 3-56.4 | Luo *et al*. 2014;  Khasawneh *et al*. 2021 |
| Phenytoin | 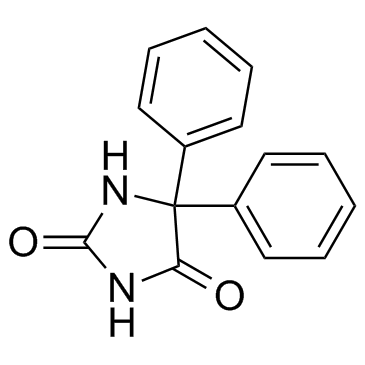 | Anti-seizure medication | 0.452 | - | 25-50 | Onesios-Barry *et al*. 2014;  Dong *et al*. 2015 |
| Primidone | 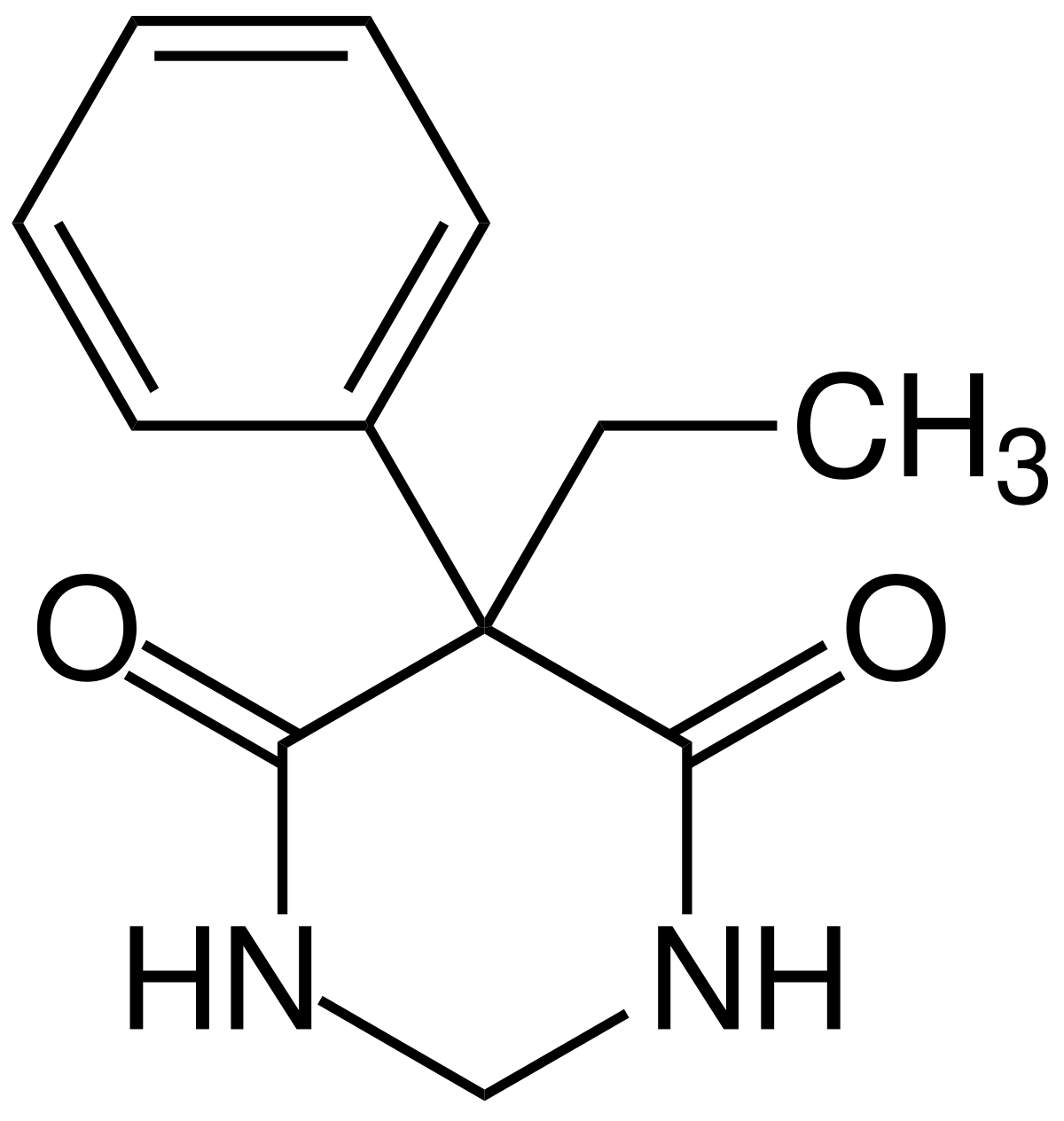 | Barbiturate | 0.517 | 0.145 | 83.5 | Kanaujiya *et al*. 2019;  Dong *et al*. 2015;  Liu *et al*. 2019 |
| Sotalol | 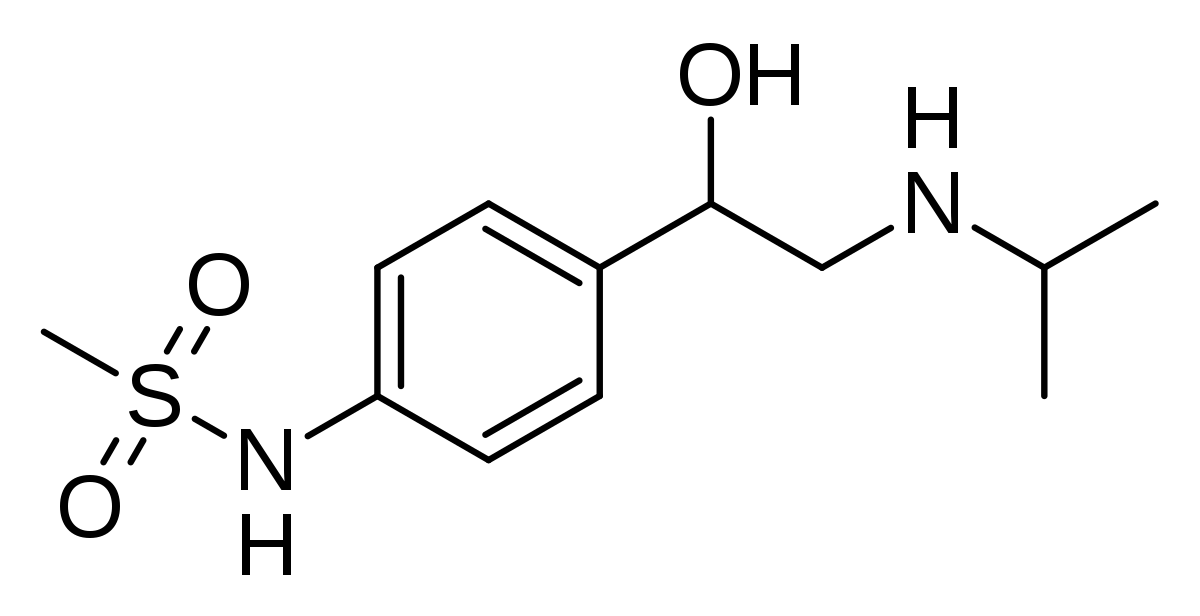 | Beta blockers | ND-0.19 | - | 55 | Sipma *et al*. 2010;  Khasawneh *et al*. 2021 |
| Sulfamethoxazole | 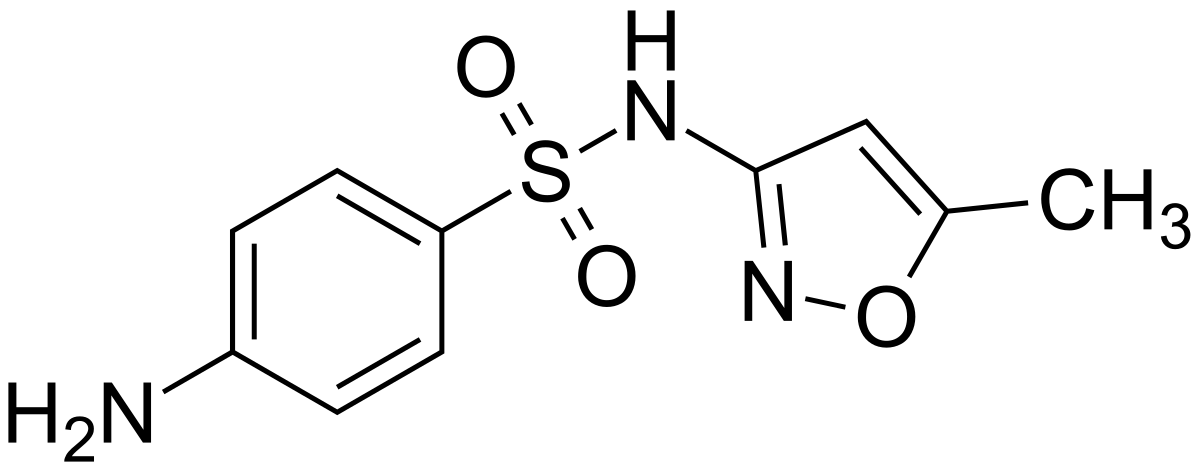 | Antibacterials | ND-54.8 | 0-74.4 | 4-88.9 | Luo *et al*. 2014;  Khasawneh *et al*. 2021  Zhou *et al*. 2022 |
| Tramadol | 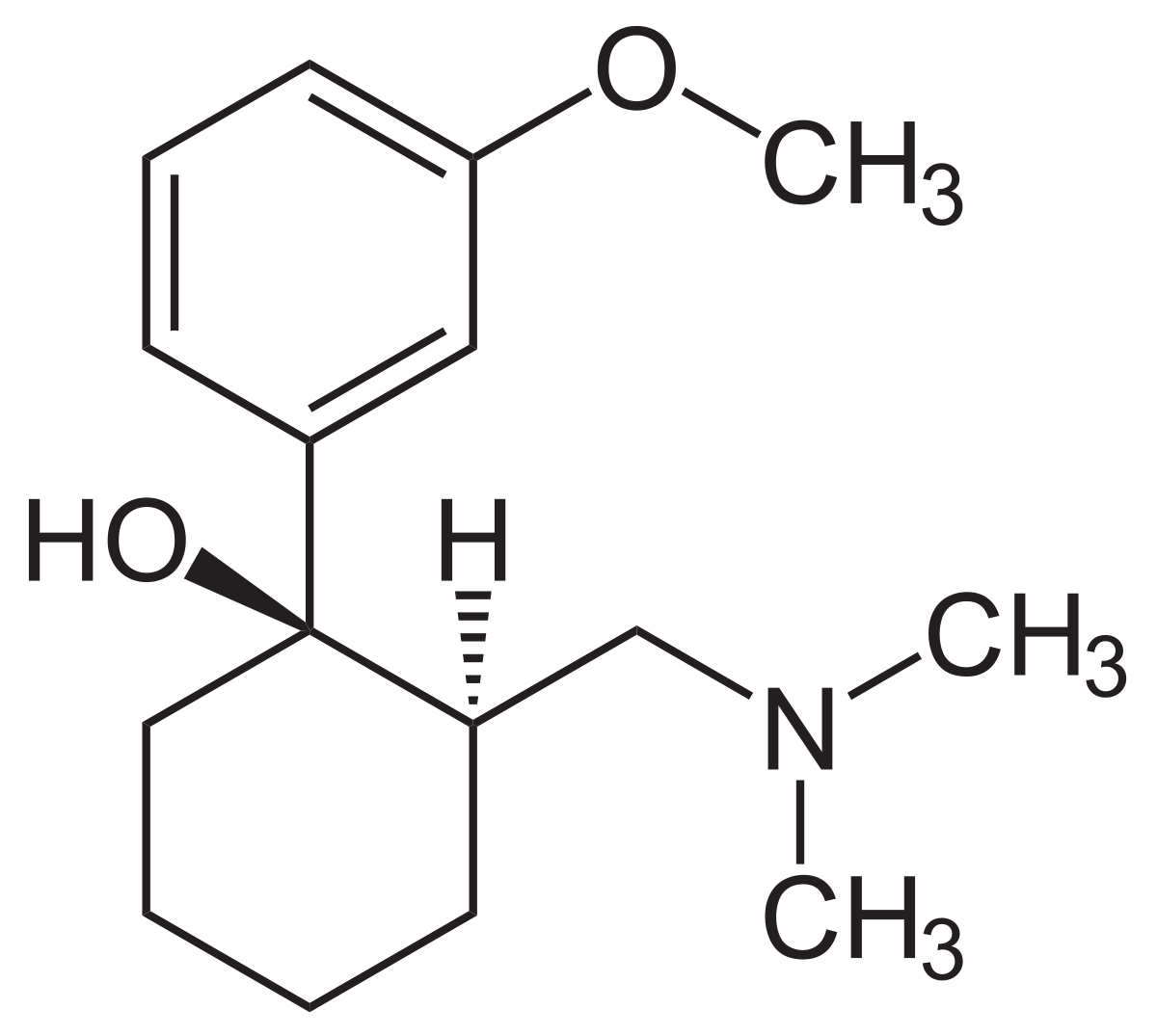 | Analgesic | 0.072-9.86 | 0.006 | 14.4 | Kanaujiya *et al*. 2019;  Khasawneh *et al*. 2021;  Long *et al*. 2023 |
| Trimethoprim | 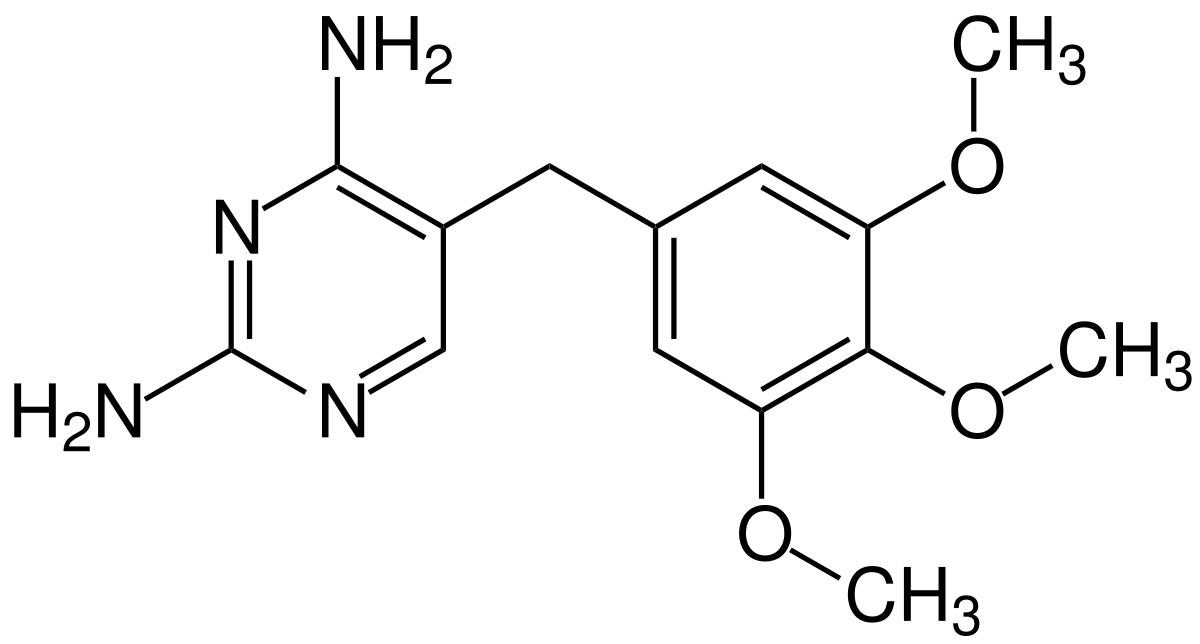 | Antibacterials | 13.6 | 2.3 | <0-81.6 | Luo *et al*. 2014;  Khasawneh *et al*. 2021;  Mostafa *et al*. 2023 |
| Venlafaxine | 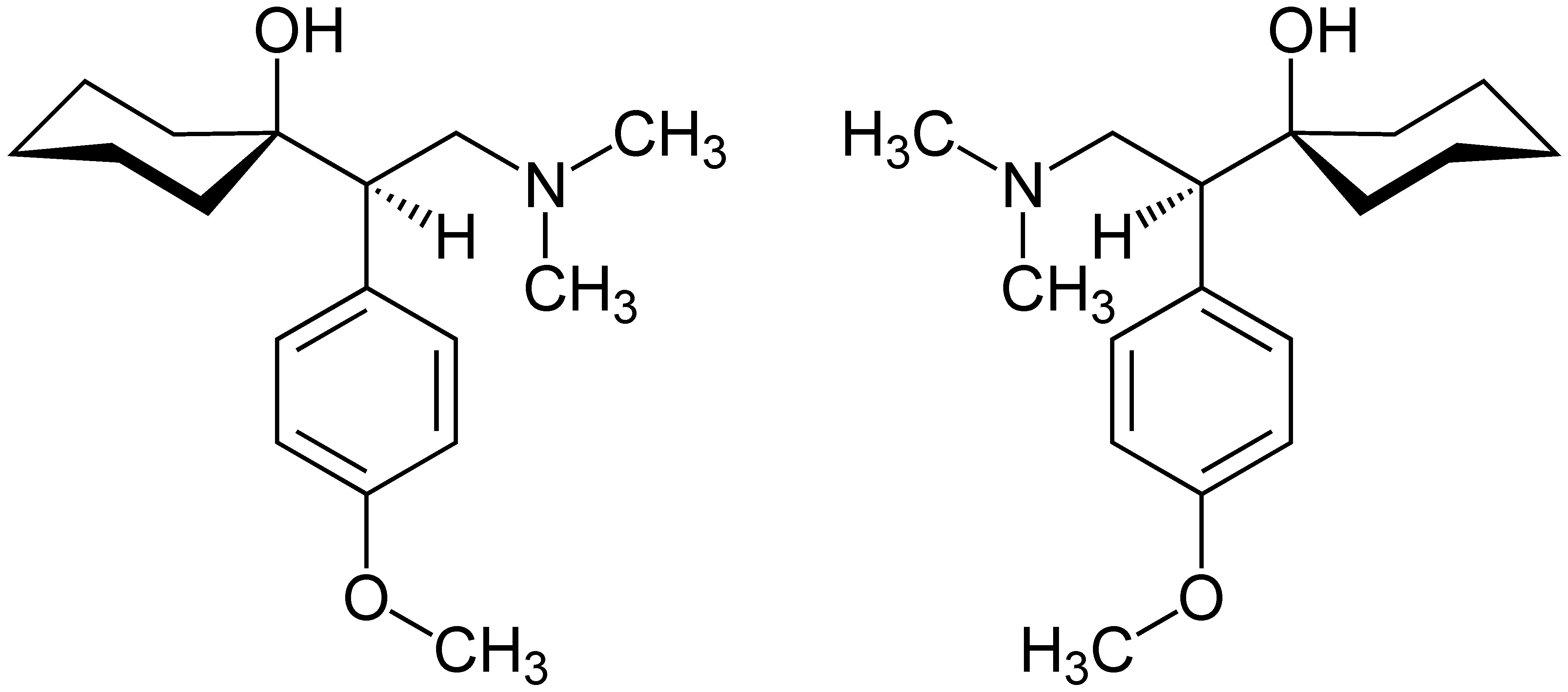 | Psychoanaleptics | ND-7.69 | 0.8 | 70 | Llorca *et al*. 2019;  Khasawneh *et al*. 2021;  Rapp-Wright *et al*. 2023 |
| 4-Formylaminoantipyrine | 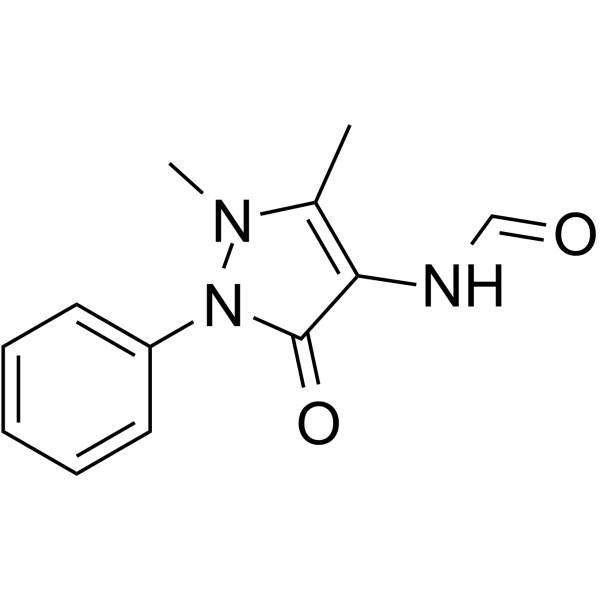 | Anti-inflammatory | ND-71 | - | 20 | Fundneider *et al*. 2021;  Zhang *et al*. 2023 |
| 4/5-Methylbenzotriazole | 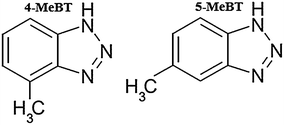 | Corrosion inhibitor | 0.4 | - | - | Kreuzig *et al*. 2021 |

**Reference**

Al Aukidy, M., Verlicchi, P., & Voulvoulis, N. (2014). A framework for the assessment of the environmental risk posed by pharmaceuticals originating from hospital effluents. Science of the Total Environment, 493, 54-64.

Almeida, B., Kjeldal, H., Lolas, I., Knudsen, A. D., Carvalho, G., Nielsen, K. L., ... & Nielsen, J. L. (2013). Quantitative proteomic analysis of ibuprofen-degrading Patulibacter sp. strain I11. Biodegradation, 24(5), 615-630.

Alotaibi, M. D., McKinley, A. J., Patterson, B. M., & Reeder, A. Y. (2015). Benzotriazoles in the aquatic environment: a review of their occurrence, toxicity, degradation and analysis. Water, Air, & Soil Pollution, 226, 1-20.

Anagnostopoulou, K., Nannou, C., Evgenidou, E., & Lambropoulou, D. A. (2023). Does climbazole instigate a threat in the environment as Persistent, Mobile and Toxic compound? Unveiling the occurrence and potential ecological risks of its phototransformation products in the water cycle. Journal of Hazardous Materials, 131854.

Assress, H. A., Nyoni, H., Mamba, B. B., & Msagati, T. A. (2020). Occurrence and risk assessment of azole antifungal drugs in water and wastewater. Ecotoxicology and environmental safety, 187, 109868.

Athanasakoglou, A., & Fenner, K. (2021). Toward Characterizing the Genetic Basis of Trace Organic Contaminant Biotransformation in Activated Sludge: The Role of Multicopper Oxidases as a Case Study. Environmental science & technology.

Bayer, A., Asner, R., Schüssler, W., Kopf, W., Weiß, K., Sengl, M., & Letzel, M. (2014). Behavior of sartans (antihypertensive drugs) in wastewater treatment plants, their occurrence and risk for the aquatic environment. Environmental Science and Pollution Research, 21(18), 10830-10839.

Bollmann, A. F., Seitz, W., Prasse, C., Lucke, T., Schulz, W., & Ternes, T. (2016). Occurrence and fate of amisulpride, sulpiride, and lamotrigine in municipal wastewater treatment plants with biological treatment and ozonation. Journal of hazardous materials, 320, 204-215.

Buerge, I. J., Buser, H. R., Kahle, M., Muller, M. D., & Poiger, T. (2009). Ubiquitous occurrence of the artificial sweetener acesulfame in the aquatic environment: an ideal chemical marker of domestic wastewater in groundwater. Environmental science & technology, 43(12), 4381-4385.

Burke, V., Schneider, L., Greskowiak, J., Zerball-van Baar, P., Sperlich, A., Dünnbier, U., & Massmann, G. (2018). Trace organic removal during river bank filtration for two types of sediment. Water, 10(12), 1736.

Chen, Y., Vymazal, J., Březinová, T., Koželuh, M., Kule, L., Huang, J., & Chen, Z. (2016). Occurrence, removal and environmental risk assessment of pharmaceuticals and personal care products in rural wastewater treatment wetlands. Science of the Total Environment, 566, 1660-1669.

Cunha, D. L., de Araujo, F. G., & Marques, M. (2017). Psychoactive drugs: occurrence in aquatic environment, analytical methods, and ecotoxicity—a review. Environmental Science and Pollution Research, 24(31), 24076-24091.

Daouk, S., Chèvre, N., Vernaz, N., Widmer, C., Daali, Y., & Fleury-Souverain, S. (2016). Dynamics of active pharmaceutical ingredients loads in a Swiss university hospital wastewaters and prediction of the related environmental risk for the aquatic ecosystems. Science of The Total Environment, 547, 244-253.

Dong, M. M., Trenholm, R., & Rosario-Ortiz, F. L. (2015). Photochemical degradation of atenolol, carbamazepine, meprobamate, phenytoin and primidone in wastewater effluents. Journal of Hazardous Materials, 282, 216-223.

Díaz-Garduño, B., Pintado-Herrera, M. G., Biel-Maeso, M., Rueda-Márquez, J. J., Lara-Martín, P. A., Perales, J. A., ... & Martín-Díaz, M. L. (2017). Environmental risk assessment of effluents as a whole emerging contaminant: Efficiency of alternative tertiary treatments for wastewater depuration. Water Research, 119, 136-149.

Faria, C. V., Ricci, B. C., Silva, A. F., Amaral, M. C., & Fonseca, F. V. (2020). Removal of micropollutants in domestic wastewater by expanded granular sludge bed membrane bioreactor. Process Safety and Environmental Protection, 136, 223-233.

Fundneider, T., Alonso, V. A., Wick, A., Albrecht, D., & Lackner, S. (2021). Implications of biological activated carbon filters for micropollutant removal in wastewater treatment. Water Research, 189, 116588.

Gimeno, O., García-Araya, J. F., Beltrán, F. J., Rivas, F. J., & Espejo, A. (2016). Removal of emerging contaminants from a primary effluent of municipal wastewater by means of sequential biological degradation-solar photocatalytic oxidation processes. Chemical Engineering Journal, 290, 12-20.

Herrmann, M., Menz, J., Olsson, O., & Kümmerer, K. (2015). Identification of phototransformation products of the antiepileptic drug gabapentin: biodegradability and initial assessment of toxicity. Water research, 85, 11-21.

Huang, Y., Deng, Y., Law, J. C. F., Yang, Y., Ding, J., Leung, K. S. Y., & Zhang, T. (2021). Acesulfame aerobic biodegradation by enriched consortia and Chelatococcus spp.: Kinetics, transformation products, and genomic characterization. Water Research, 202, 117454.

Kahl, S., Kleinsteuber, S., Nivala, J., van Afferden, M., & Reemtsma, T. (2018). Emerging biodegradation of the previously persistent artificial sweetener acesulfame in biological wastewater treatment. Environmental science & technology, 52(5), 2717-2725.

Kanaujiya, D. K., Paul, T., Sinharoy, A., & Pakshirajan, K. (2019). Biological Treatment Processes for the Removal of Organic Micropollutants from Wastewater: a Review. Current pollution reports, 5(3), 112-128.

Khare, A., Jadhao, P., Kawre, S., Kanade, G., Patil, M., Vaidya, A. N., & Kumar, A. R. (2023). Occurrence, spatio-temporal variation and ecological risk assessment of benzotriazole ultraviolet stabilizers (BUVs) in water and sediment of rivers in central India. Science of The Total Environment, 882, 163381.

Khasawneh, O. F. S., & Palaniandy, P. (2021). Occurrence and removal of pharmaceuticals in wastewater treatment plants. Process Safety and Environmental Protection, 150, 532-556.

Kim, S. D., Cho, J., Kim, I. S., Vanderford, B. J., & Snyder, S. A. (2007). Occurrence and removal of pharmaceuticals and endocrine disruptors in South Korean surface, drinking, and waste waters. Water research, 41(5), 1013-1021.

Kim, J. Y., Jeon, J., & Kim, S. D. (2023). Prioritization of pharmaceuticals and personal care products in the surface waters of Korea: Application of an optimized risk-based methods. Ecotoxicology and Environmental Safety, 259, 115024.

Kjeldal, H., Zhou, N. A., Wissenbach, D. K., von Bergen, M., Gough, H. L., & Nielsen, J. L. (2016). Genomic, proteomic, and metabolite characterization of gemfibrozil-degrading organism Bacillus sp. GeD10. Environmental Science & Technology, 50(2), 744-755.

Kreuzig, R., Haller-Jans, J., Bischoff, C., Leppin, J., Germer, J., Mohr, M., ... & Dockhorn, T. (2021). Reclaimed water driven lettuce cultivation in a hydroponic system: the need of micropollutant removal by advanced wastewater treatment. Environmental Science and Pollution Research, 28(36), 50052-50062.

Kucharski, D., Nałęcz-Jawecki, G., Drzewicz, P., Skowronek, A., Mianowicz, K., Strzelecka, A., & Giebułtowicz, J. (2022). The assessment of environmental risk related to the occurrence of pharmaceuticals in bottom sediments of the Odra River estuary (SW Baltic Sea). Science of The Total Environment, 828, 154446.

Liu, M., Yin, H., & Wu, Q. (2019). Occurrence and health risk assessment of pharmaceutical and personal care products (PPCPs) in tap water of Shanghai. Ecotoxicology and Environmental Safety, 183, 109497.

Llorca, M., Castellet-Rovira, F., Farré, M. J., Jaén-Gil, A., Martínez-Alonso, M., Rodríguez-Mozaz, S., ... & Barceló, D. (2019). Fungal biodegradation of the N-nitrosodimethylamine precursors venlafaxine and O-desmethylvenlafaxine in water. Environmental Pollution, 246, 346-356.

Long, B. M., Harriage, S., Schultz, N. L., Sherman, C. D., & Thomas, M. (2023). Pharmaceutical pollution in marine waters and benthic flora of the southern Australian coastline. Environmental Chemistry, 19(6), 375-384.

Luo, Y., Guo, W., Ngo, H. H., Nghiem, L. D., Hai, F. I., Zhang, J., ... & Wang, X. C. (2014). A review on the occurrence of micropollutants in the aquatic environment and their fate and removal during wastewater treatment. Science of the total environment, 473, 619-641.

Monapathi, M. E., Oguegbulu, J. C., Adogo, L., Klink, M., Okoli, B., Mtunzi, F., & Modise, J. S. (2021). Pharmaceutical pollution: azole antifungal drugs and resistance of opportunistic pathogenic yeasts in wastewater and environmental water. Applied and Environmental Soil Science, 2021, 1-11.

Mostafa, A., Shaaban, H., Alqarni, A., Al-Ansari, R., Alrashidi, A., Al-Sultan, F., ... & Aga, O. (2023). Multi-class determination of pharmaceuticals as emerging contaminants in wastewater from Eastern Province, Saudi Arabia using eco-friendly SPE-UHPLC-MS/MS: Occurrence, removal and environmental risk assessment. Microchemical Journal, 187, 108453.

Onesios-Barry, K. M., Berry, D., Proescher, J. B., Sivakumar, I. A., & Bouwer, E. J. (2014). Removal of pharmaceuticals and personal care products during water recycling: microbial community structure and effects of substrate concentration. Applied and environmental microbiology, 80(8), 2440-2450.

Pan, C. G., Peng, F. J., & Ying, G. G. (2018). Removal, biotransformation and toxicity variations of climbazole by freshwater algae Scenedesmus obliquus. Environmental Pollution, 240, 534-540.

Rapp-Wright, H., Regan, F., White, B., & Barron, L. P. (2023). A year-long study of the occurrence and risk of over 140 contaminants of emerging concern in wastewater influent, effluent and receiving waters in the Republic of Ireland. Science of The Total Environment, 860, 160379.

Schulz, M., Löffler, D., Wagner, M., & Ternes, T. A. (2008). Transformation of the X-ray contrast medium iopromide in soil and biological wastewater treatment. Environmental science & technology, 42(19), 7207-7217.

Selak, A., Reberski, J. L., Klobučar, G., & Grčić, I. (2022). Ecotoxicological aspects related to the occurrence of emerging contaminants in the Dinaric karst aquifer of Jadro and Žrnovnica springs. Science of the Total Environment, 825, 153827.

Shen, G., Lei, S., Li, H., Yu, Q., Wu, G., Shi, Y., ... & Geng, J. (2023). Occurrence and removal of four artificial sweeteners in wastewater treatment plants of China. Environmental Science: Processes & Impacts, 25(1), 75-84.

Singh, A., Saidulu, D., Gupta, A. K., & Kubsad, V. (2022). Occurrence and fate of antidepressants in the aquatic environment: Insights into toxicological effects on the aquatic life, analytical methods, and removal techniques. Journal of Environmental Chemical Engineering, 109012.

Sipma, J., Osuna, B., Collado, N., Monclús, H., Ferrero, G., Comas, J., & Rodriguez-Roda, I. (2010). Comparison of removal of pharmaceuticals in MBR and activated sludge systems. Desalination, 250(2), 653-659.

Suarez, S., Lema, J. M., & Omil, F. (2010). Removal of pharmaceutical and personal care products (PPCPs) under nitrifying and denitrifying conditions. Water research, 44(10), 3214-3224.

Torresi, E., Tang, K., Deng, J., Sund, C., Smets, B. F., Christensson, M., & Andersen, H. R. (2019). Removal of micropollutants during biological phosphorus removal: Impact of redox conditions in MBBR. Science of the total environment, 663, 496-506.

Yi, M., Sheng, Q., Lv, Z., & Lu, H. (2022). Novel pathway and acetate-facilitated complete atenolol degradation by Hydrogenophaga sp. YM1 isolated from activated sludge. Science of The Total Environment, 810, 152218.

Zhang, Y., Wang, J., Cui, H., Gao, S., Ye, L., Li, Z., ... & Liang, B. (2023). Environmental occurrence, risk, and removal strategies of pyrazolones: A critical review. Journal of Hazardous Materials, 132471.

Zhou, J., Wang, D., Ju, F., Hu, W., Liang, J., Bai, Y., ... & Qu, J. (2022). Profiling microbial removal of micropollutants in sand filters: Biotransformation pathways and associated bacteria. Journal of Hazardous Materials, 423, 127167.

Żur, J., Piński, A., Wojcieszyńska, D., Smułek, W., & Guzik, U. (2020). Diclofenac degradation—enzymes, genetic background and cellular alterations triggered in diclofenac-metabolizing strain Pseudomonas moorei KB4. International journal of molecular sciences, 21(18), 6786.
