# Supplementary material for: Establishment of microbial model communities capable of removing trace organic chemicals for biotransformation mechanisms research
Source: Microb Cell Fact. 2023 Dec 2;22:245. doi: 10.1186/s12934-023-02252-6 (PMC10693053; doi:10.1186/s12934-023-02252-6)
Supplement: Supplementary file 2 — Additional file 2: Figure S1. Cell counts after 21 days incubation in six stages. Figure S2. Cell counts of communities growing from different diluted cell numbers (below growth threshold) in the (a) pre-adaptation and (b) non-adaptation group after 21 days incubation, n = 48. Figure S3. Heatmap illustrating 27 TOrCs removal efficiencies by thirty microbial communities. The color legend represents the removal percentage. Figure S4. Taxonomic composition of 11 model communities selected after TOrC removal performance assessment at the genus level. Numbers in the pie chart represent the OTUs belonging to each genus. Figure S5. Comparison of microbial structure between pre- and non-adapted inocula at the family level. Figure S6. Thirty model communities’ frequency on simultaneously removed TOrC number. The removal cutoff is 20%. [file 12934_2023_2252_MOESM2_ESM.docx]

Establishment of microbial model communities capable of removing trace organic chemicals for biotransformation mechanisms research

(Supplementary Material)

Figure S1. Cell counts after 21 days incubation in six stages.

Figure S2. Cell counts of communities growing from different diluted cell numbers (below growth threshold) in the (a) pre-adaptation and (b) non-adaptation group after 21 days incubation, n = 48.


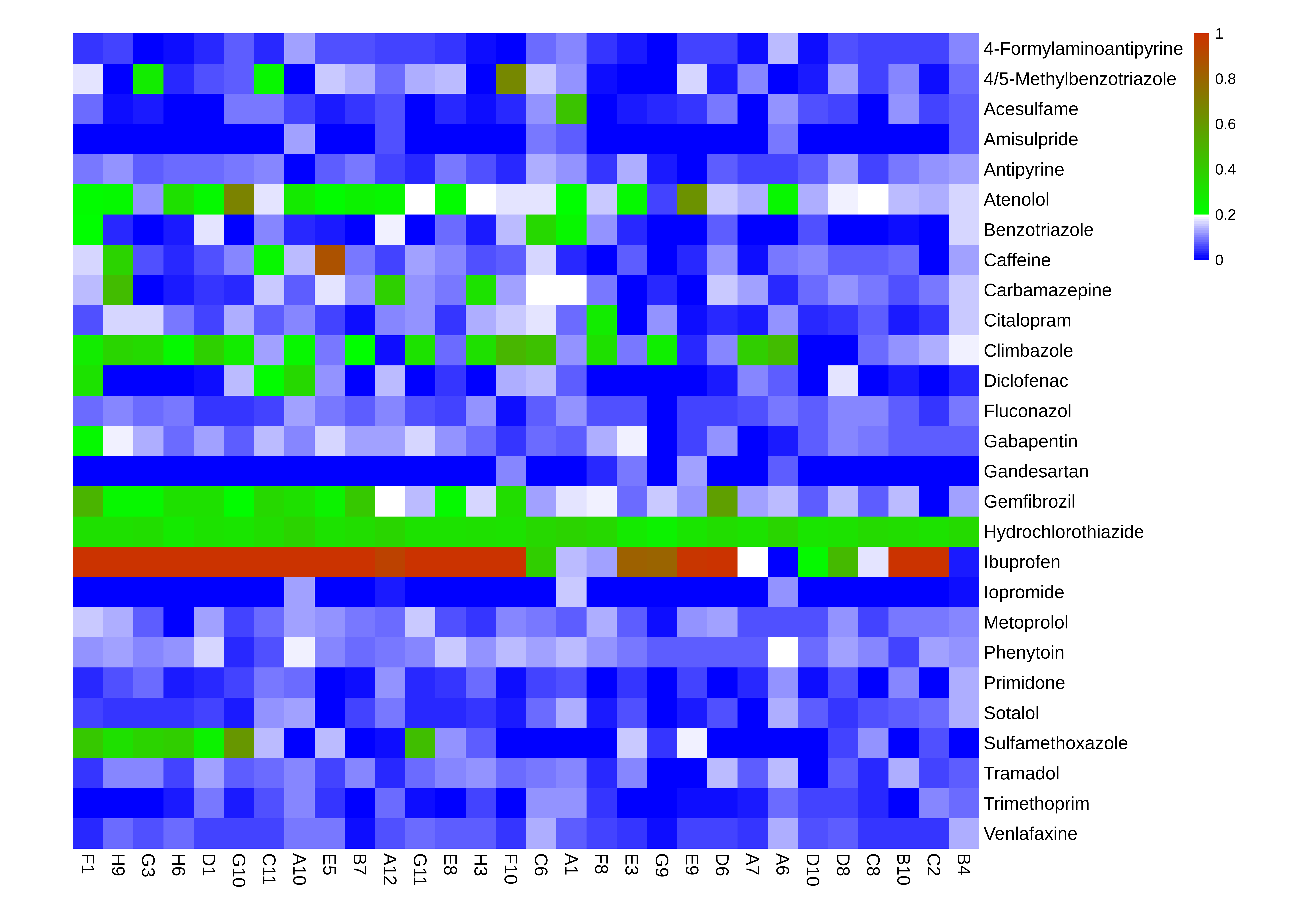


Figure S3. Heatmap illustrating 27 TOrCs removal efficiencies by thirty microbial communities. The color legend represents the removal percentage.

Figure S4. Taxonomic composition of 11 model communities selected after TOrC removal performance assessment at the genus level. Numbers in the pie chart represent the OTUs belonging to each genus.


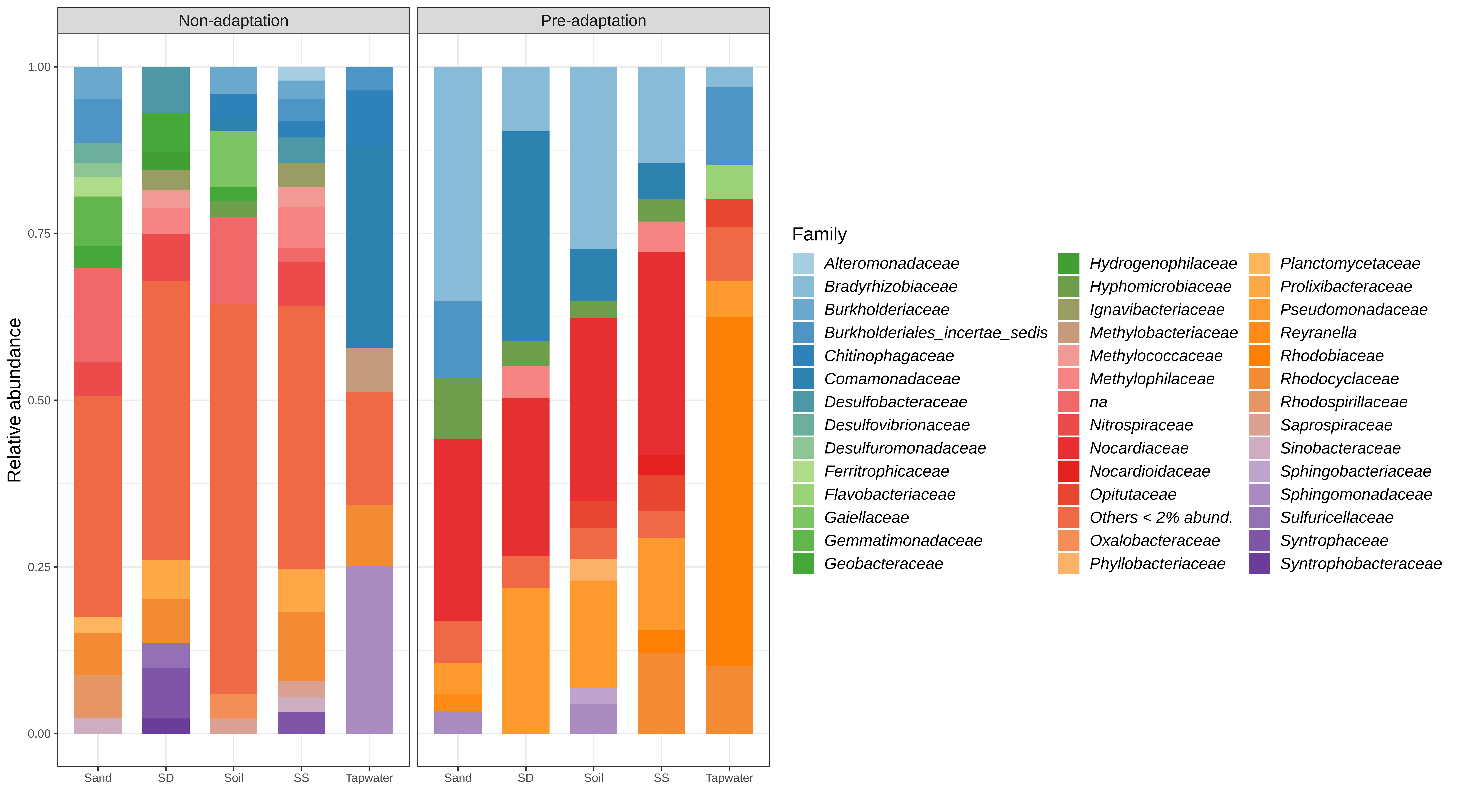


Figure S5. Comparison of microbial structure between pre- and non-adapted inocula at the family level.


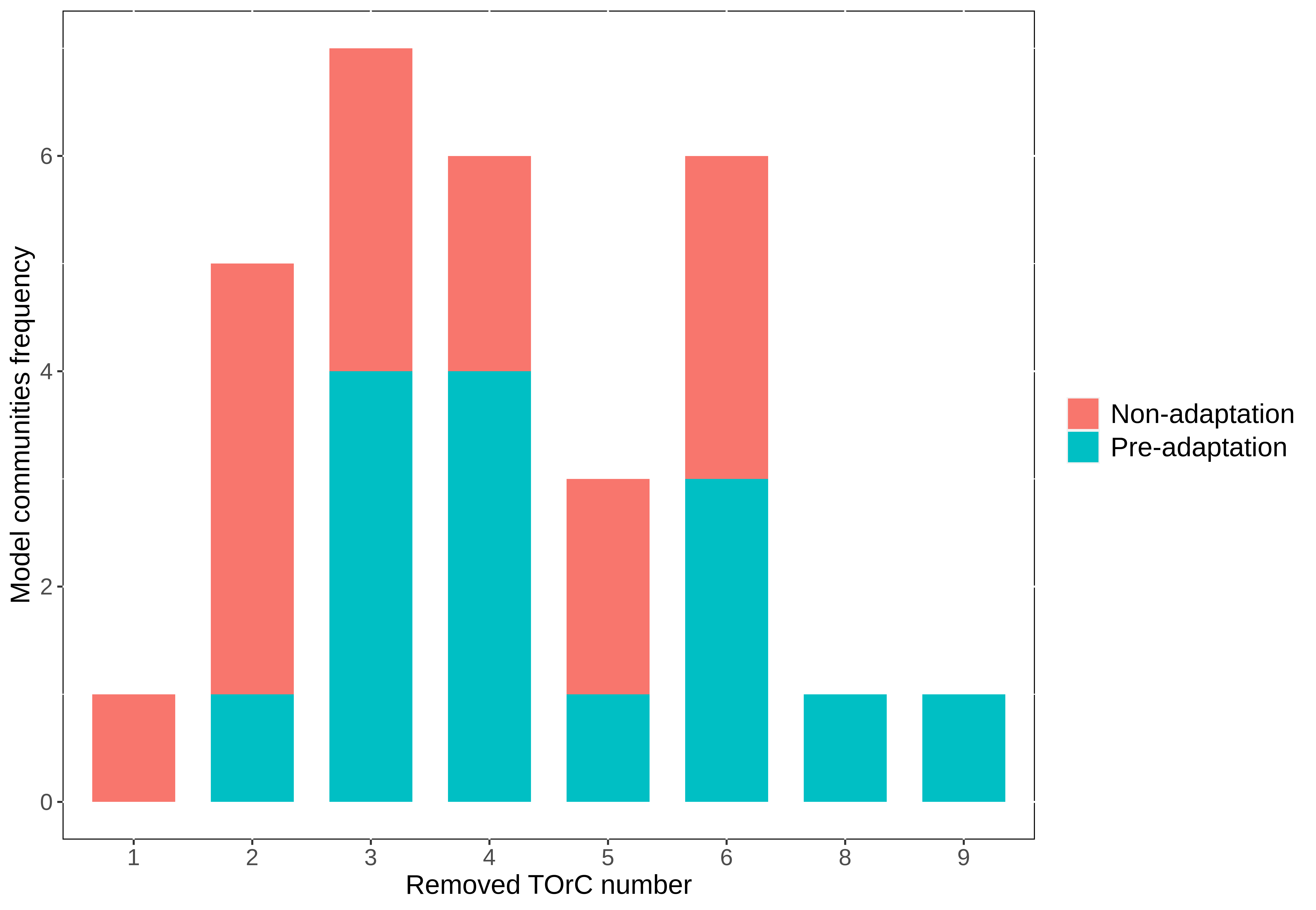


Figure S6. Thirty model communities’ frequency on simultaneously removed TOrC number. The removal cutoff is 20%.
